# 2,3,7,8 Tetrachlorodibenzo-*p*-dioxin-induced RNA abundance changes identify *Ackr3*, *Col18a1*, *Cyb5a* and *Glud1* as candidate mediators of toxicity

**DOI:** 10.1007/s00204-016-1720-0

**Published:** 2016-04-30

**Authors:** John D. Watson, Stephenie D. Prokopec, Ashley B. Smith, Allan B. Okey, Raimo Pohjanvirta, Paul C. Boutros

**Affiliations:** 1 Informatics and Bio-computing Program, MaRS Centre, Ontario Institute for Cancer Research, 661 University Avenue, Suite 510, Toronto, ON M5G 0A3 Canada; 2 Department of Pharmacology and Toxicology, University of Toronto, Toronto, Canada; 3 Laboratory of Toxicology, National Institute for Health and Welfare, Kuopio, Finland; 4 Department of Food Hygiene and Environmental Health, University of Helsinki, Helsinki, Finland; 5 Department of Medical Biophysics, University of Toronto, Toronto, Canada

**Keywords:** Aryl hydrocarbon receptor, 2,3,7,8 Tetrachlorodibenzo-p-dioxin, TCDD, mRNA abundance, NanoString, Toxicity

## Abstract

2,3,7,8 Tetrachlorodibenzo-*p*-dioxin (TCDD) is an aromatic, long-lived environmental contaminant. While the pathogenesis of TCDD-induced toxicity is poorly understood, it has been shown that the aryl hydrocarbon receptor (AHR) is required. However, the specific transcriptomic changes that lead to toxic outcomes have not yet been identified. We previously identified a panel of 33 genes that respond to TCDD treatment in two TCDD-sensitive rodent species. To identify genes involved in the onset of hepatic toxicity, we explored 25 of these in-depth using liver from two rat strains: the TCDD-resistant Han/Wistar (H/W) and the TCDD-sensitive Long–Evans (L–E). Time course and dose–response analyses of mRNA abundance following TCDD insult indicate that eight genes are similarly regulated in livers of both strains of rat, suggesting that they are not central to the severe L–E-specific TCDD-induced toxicities. The remaining 17 genes exhibited various divergent mRNA abundances between L–E and H/W strains after TCDD treatment. Several genes displayed a biphasic response where the initial response to TCDD treatment was followed by a secondary response, usually of larger magnitude in L–E liver. This secondary response was most often an exaggeration of the original TCDD-induced response. Only cytochrome b5 type A (microsomal) (*Cyb5a*) had equivalent TCDD sensitivity to the prototypic AHR-responsive cytochrome P450, family 1, subfamily a, polypeptide 1 (*Cyp1a1*), while six genes were less sensitive. Four genes showed an early inter-strain difference that was sustained throughout most of the time course (atypical chemokine receptor 3 (*Ackr3*), collagen, type XVIII, alpha 1 (*Col18a1*), *Cyb5a* and glutamate dehydrogenase 1 (*Glud1*)), and of those genes examined in this study, are most likely to represent genes involved in the pathogenesis of TCDD-induced hepatotoxicity in L–E rats.

**Electronic supplementary material:**

The online version of this article (doi:10.1007/s00204-016-1720-0) contains supplementary material, which is available to authorized users.

## Introduction

The environmental contaminant 2,3,7,8 tetrachlorodibenzo-*p*-dioxin (TCDD) is a highly stable aromatic compound that causes a wide variety of toxic phenotypes. Specific toxic effects vary across species, but in mammals can include hepatic toxicity, chloracne, teratogenic effects, thymic atrophy, immune dysregulation, rapid weight loss (known as wasting syndrome) and cancer (Birnbaum and Tuomisto 2000; Pohjanvirta and Tuomisto 1994; White and Birnbaum 2009). TCDD is highly lipophilic and poorly metabolized, and thus bio-accumulates within fat stores of animals higher up the food chain and represents a long-term, cumulative source of toxicity (Domingo and Bocio 2007; Sinkkonen and Paasivirta 2000; van Birgelen and van den Berg 2000).

While TCDD exposure leads to toxicity in most vertebrate species, there is a high degree of variation in susceptibility, both between species and within species. Notably, the Han/Wistar (*Kuopio*) rat (H/W) is exceptionally resistant to TCDD-induced toxicities (LD_50_ above 9600 μg/kg), while guinea pigs are at least 10,000-fold more sensitive, with an LD_50_ of 1–2 μg/kg (Pohjanvirta and Tuomisto 1994; Pohjanvirta et al. 1999). Most mammalian species fall between these extremes: commonly used experimental models such as the Long–Evans (*Turku/*AB; L–E) rat and C57BL/6 mouse have intermediate sensitivity (LD_50_ of 17.7 and ~182 μg/kg for male animals, respectively) (Pohjanvirta and Tuomisto 1994). In addition to variation in sensitivity among species, the specific tissues and organ systems affected by TCDD ingestion vary widely among species. In humans, the most obvious outcome of exposure to high doses of TCDD is chloracne, whereas wasting syndrome and delayed death are most notable in rodents (Sorg et al. 2009; Sweeney and Mocarelli 2000; Tuomisto et al. 1995). Studies of human populations exposed to TCDD and related congeners (resulting from industrial accidents and food contamination) have implicated TCDD as a human carcinogen, although this finding is widely debated due to confounding factors (Consonni et al. 2008; Pesatori et al. 2009; Tuomisto and Tuomisto 2012; US-EPA 2003).

Despite wide variation in sensitivity and differing manifestations of toxicity among species, the toxic effects of TCDD have been mainly attributed to activation of the aryl hydrocarbon receptor (AHR) (Okey 2007). The AHR is a ligand-activated transcription factor of the Per-Arnt-Sim family and alters transcription of numerous genes (Kewley et al. 2004). Upon ligand binding, the AHR is translocated into the cell nucleus where it heterodimerizes with the aryl hydrocarbon nuclear translocator (ARNT), subsequently binding to AHR response elements (AHREs) in upstream regulatory regions of target genes such as *Cyp1a1* (Lindebro et al. 1995). Studies in mice have shown that mutations which reduce the affinity of AHR for TCDD correlate with a reduction in toxic outcome (Birnbaum et al. 1990; Okey et al. 1989). In the H/W rat, a point mutation that alters the AHR transactivation domain via alternative splicing imparts great resistance to TCDD-induced toxicities (Pohjanvirta et al. 1998; Simanainen et al. 2002). It is interesting that the resistance of H/W rats to the toxic effects of TCDD exposure occurs despite the variant H/W AHR maintaining the ability to regulate transcriptional changes in numerous “AHR-core” genes (such as *Cyp1a1*) in a manner similar to TCDD-sensitive L–E rats (Moffat et al. 2010; Simanainen et al. 2002). Responses that are conserved between sensitive and resistant rat strains are termed type I, while responses that are enhanced in or exclusive to TCDD-sensitive L–E rats are termed type II responses (Simanainen et al. 2002, 2003).

It has also been shown that heterodimerization of the AHR with ARNT is required for TCDD-induced toxicity in mouse liver (Nukaya et al. 2010), and that mice hypomorphic for ARNT are resistant to TCDD-induced effects (Walisser et al. 2004a, b). However, the clearest evidence that TCDD toxicity is AHR dependent comes from studies of AHR knockout mice. Mice lacking an AHR do not suffer the toxic effects of TCDD (Bunger et al. 2003; Fernandez-Salguero et al. 1996; Mimura et al. 1997; Vorderstrasse et al. 2001). While some studies have identified non-genomic pathways leading to metabolic alterations (Li et al. 2010; Matsumura 2009), the studies discussed above indicate that DNA binding of the AHR:ARNT heterodimer is required to mediate the major toxic outcomes of dioxin exposure.

Identification of the key transcriptional changes that lead to toxicity in laboratory species has been difficult. Activation of the AHR alters the abundance of hundreds to thousands of different mRNAs (Boutros et al. 2008; Boverhof et al. 2006; Forgacs et al. 2013; Sato et al. 2008; Tijet et al. 2006). While a core set of genes is affected across a wide biological spectrum (termed “AHR-core” genes), the majority of transcriptomic responses appear to depend upon the species, strain, tissue and cell type (Boutros et al. 2008; Boverhof et al. 2006; Carlson et al. 2009; Puga et al. 2004; Watson et al. 2013). In general, “AHR-core” genes are involved in pathways for detoxification (*e.g.* *Cyp1a1*), oxidative stress [e.g. nuclear factor, erythroid 2-like 2 (*Nfe2l2*)] and negative feedback regulation [e.g. aryl hydrocarbon receptor repressor (*Ahrr*)]. This complex variation in transcriptional response is coupled to a large degree of intra-species and even intra-strain variation in the pattern of phenotypic responses to TCDD treatment described above. Indeed, the large intra-species variability in TCDD-induced changes is a reflection of large differences in the basal transcriptome across strains of rats (Yao et al. 2012) and mice (Pritchard et al. 2006).

Fortunately, these variations also provide a tool that can be used to identify genes involved in toxicity. Recent studies by our group (Boutros et al. 2008) and others (Boverhof et al. 2006) have compared mouse and rat, two TCDD-sensitive laboratory animals that have similar phenotypic responses to TCDD. Comparison of hepatic mRNA abundance changes following TCDD treatment identified several genes which were dysregulated in both species and may be involved in the onset of liver toxicity. While each of these studies identified 33 genes that were similarly regulated in both mouse and rat, only three genes [*Cyp1a1*, NAD(P)H dehydrogenase, quinone 1 (*Nqo1*) and glutamate dehydrogenase 1 (*Glud1*)] were identified in both studies. Continuing from the detailed analysis of eight “AHR-core” genes (Watson et al. 2013), the remaining 25 TCDD-responsive genes identified in our rat–mouse comparison are analysed in detail here. We compare changes in hepatic mRNA abundance between the TCDD-resistant H/W rat and the TCDD-sensitive L–E rat. By identifying genes whose expression differs between dioxin-sensitive and dioxin-resistant rats following TCDD exposure, we have identified candidate regulators of type II phenotypic responses.

## Methods and materials

### Animal handling

Samples used in this study were the same as previously described (Watson et al. 2013). This manuscript does not contain any clinical studies or patient data. Study plans were approved by the Kuopio Provincial Government and the Animal Experiment Committee of the University of Kuopio. Briefly, four experimental (TCDD treated) rats were used for each dose studied (0.001, 0.01, 0.1, 1, 10, 50, 100, 1000 or 3000 μg/kg, Fig. S1), and livers were harvested 19-h post-gavage with TCDD in corn oil. The time course study animals were treated with a single 100 μg/kg dose of TCDD in corn oil, and the liver was harvested at the appropriate times following treatment; L–E animals were harvested at 3-, 6-, 10-, 19-, 96- and 240-h post-TCDD treatment (*n* = 4, 4, 4, 4, 4, 5, respectively), and H/W animals were harvested at 1.5, 3, 6, 10, 19, 96, 240 and 384 h after TCDD treatment (*n* = 3, 4, 4, 4, 4, 5, 5, 4, respectively). In addition, animals treated by gavage with corn oil vehicle were harvested at several time points as controls [L–E: 19, 96 and 240 h (*n* = 7, 4, 5, respectively), H/W: 1.5, 19, 96, 240 and 384 h (*n* = 3, 7, 5, 5, 4, respectively), Fig. S1]. Animal weights are reported in File S1. ARRIVE guidelines for reporting animal experimentation were followed (Kilkenny et al. 2010) as outlined in the ARRIVE checklist (File S2).

### RNA isolation

RNA was extracted from rat liver using RNeasy Mini kits (Qiagen, Mississauga, Canada) following the manufacturer’s recommended protocol. RNA was quantified using a NanoDrop spectrophotometer, and the integrity of the RNA was verified by electrophoresis on an Agilent 2100 Bioanalyzer, using RNA Nano 6000 total RNA assays. Only RNA samples with an RNA integrity number (RIN) greater than 8.5 were used in downstream analyses. RIN numbers are available in Watson et al. (2013), as Supplementary File 1.

### RNA analysis

RNA was diluted to a concentration of 50 ng/µl, and 50 µl of each sample was loaded into one well of a 96-well plate and sent to the UHN Microarray Centre (Toronto, ON) on dry ice for analysis on a NanoString nCounter. Desired mRNA targets were submitted in advance, and probes were designed and synthesized by NanoString prior to RNA analysis. Probes were verified by BLAST analysis (Johnson et al. 2008), searching the *Rattus norvegicus* nr/nt database to ensure that each identified a single gene. The CodeSet (the multiplexed collection of 54 distinct probes) used is provided in File S2. All raw and pre-processed data and the CodeSet have been deposited in the NCBI’s Gene Expression Omnibus (Edgar et al. 2002) as GSE43251. Each sample was analysed in a separate hybridization reaction containing the entire CodeSet, and the NanoString data were pre-processed as previously described (Watson et al. 2013). The NanoStringNorm (version 0.9.4) package, designed for use in the R statistical environment, provides all pre-processing methods for NanoString data (Waggott et al. 2011). Since time-matched vehicle controls were not available for all time points, the 19-h vehicle control was used as the basal level for subsequent analyses. Statistical analysis indicated that use of the 19-h control instead of the available time-matched controls did not significantly alter the results (Supplementary Fig. 3 of Watson et al. 2013).

### Statistical analysis

Data were analysed in the R statistical environment (version 3.2.1) using unpaired Student’s *t* tests to compare strains, doses and time points (Ihaka and Gentleman 1996). *p* values were false discovery rate corrected (*p*_adjusted_) to adjust for multiple testing (Storey and Tibshirani 2003). ED_50_ values with 90 % confidence intervals were determined by fitting response curves using a four-parameter log-logistic model ($f(x) = c + [\{ d - c\} /1 + \exp (b(\log (x) - \tilde{e}))]$), where *b* = slope at the inflection point, *c* = lower limit, *d* = upper limit and $\tilde{e} = \log (ED_{50} )$) using the R package drc (version 2.5-12). ED_50_ with 90 % confidence intervals was determined by drc as part of the curve fitting. Differences in ED_50_ parameter values were determined between strains and *p* values generated by means of approximate *t* tests (Ritz and Streibig 2005). Data were visualized using the lattice (version 0.20-33) and latticeExtra (version 0.6-26) packages via the BPG package (P’ng et al. submitted; version 5.3.4). All error bars represent standard error of the mean. ED_50_ values were compared using inferential confidence intervals with Δ = 2 × ED_50_ 90 % confidence range for *Cyp1a1* to determine whether any gene(s) had an ED_50_ value statistically equivalent to *Cyp1a1* (Beckstead 2008; Tryon and Lewis 2008).

## Results

We previously identified 33 genes that may be involved in the onset of TCDD toxicity, having changes in liver mRNA abundance that occur in common between two rodent species that display similar phenotypic responses to TCDD (Boutros et al. 2008). The goal of our current study was to validate and prioritize candidate genes for subsequent mechanistic analysis. We chose to examine the less-studied TCDD-responsive genes by excluding the well-documented “AHR-core” genes. Here, we compare changes in hepatic mRNA abundances following TCDD treatment of TCDD-sensitive L–E rats with TCDD-resistant H/W rats. These rat strains differ widely in their phenotypic response to TCDD (Pohjanvirta et al. 1999). We therefore hypothesize that genes displaying conserved responses in TCDD-sensitive L–E rats and C57BL/6 mice but that demonstrate differential expression patterns between L–E and TCDD-resistant H/W rats play a role in the onset of toxicity.

### Time course analysis

**Fig. 1:**
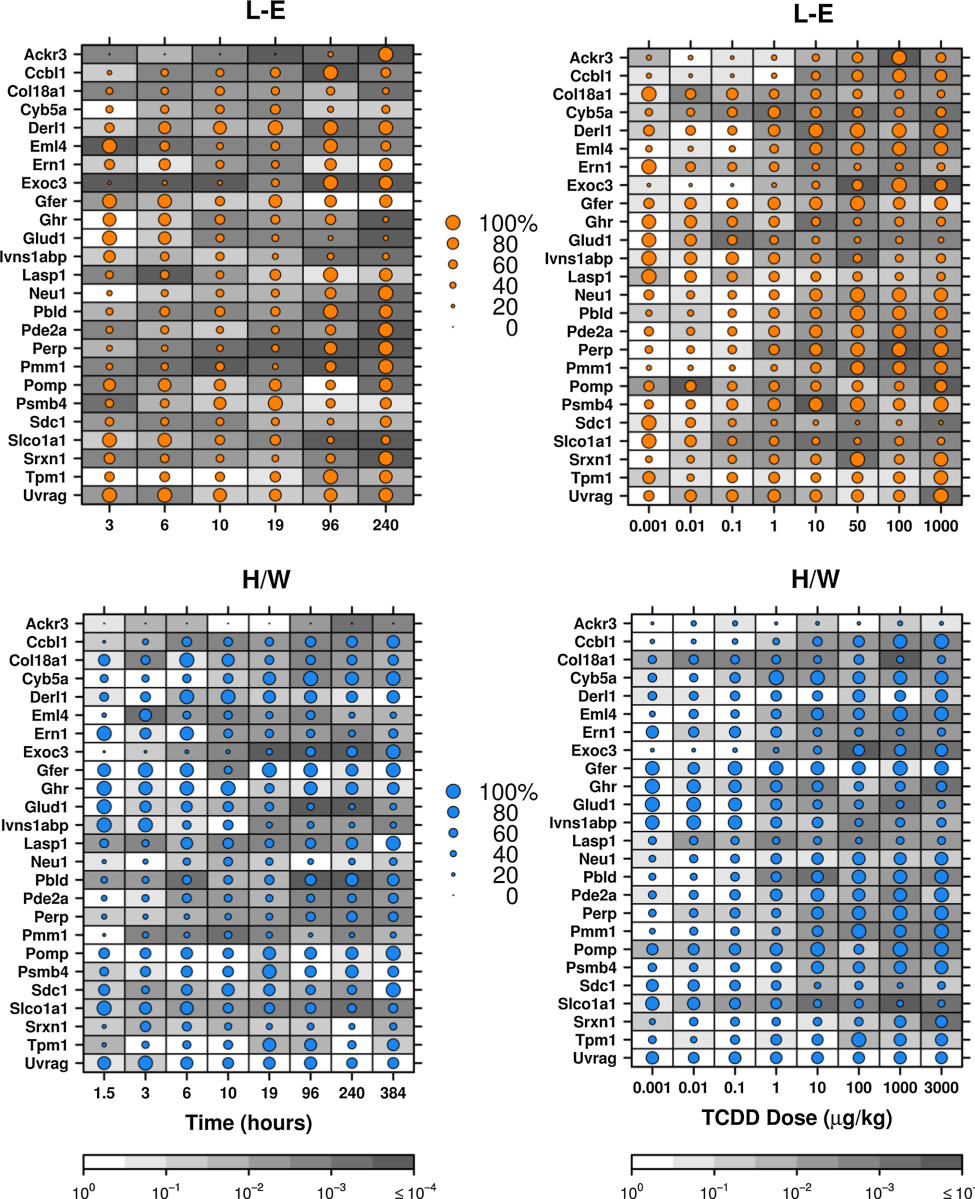
Summary of transcriptional responses to TCDD exposure. Summary of mRNA abundance changes of all examined genes following TCDD treatment with animals evaluated along either a time course (a) or dose–response (b) experiment. *Dot size* Magnitude of change as a per cent of the maximal normalized expression level for that gene in either H/W or L–E rat (whichever strain has the highest expression level) to allow for direct comparison between strains. *Shading of individual squares* represents the FDR-adjusted *p* value for an unpaired Student’s *t* test comparing TCDD-induced expression to the 19-h vehicle control for each strain. Differences from vehicle controls were considered significant if two consecutive points in the time course (normalized expression levels, not fold change) were statistically significant at a *p*_adjusted_ < 0.10, resulting in an FDR-adjusted joint probability of <0.01. H/W values are represented by blue circles, while L–E are represented by *orange circles* (colour figure online)

**Fig. 2:**
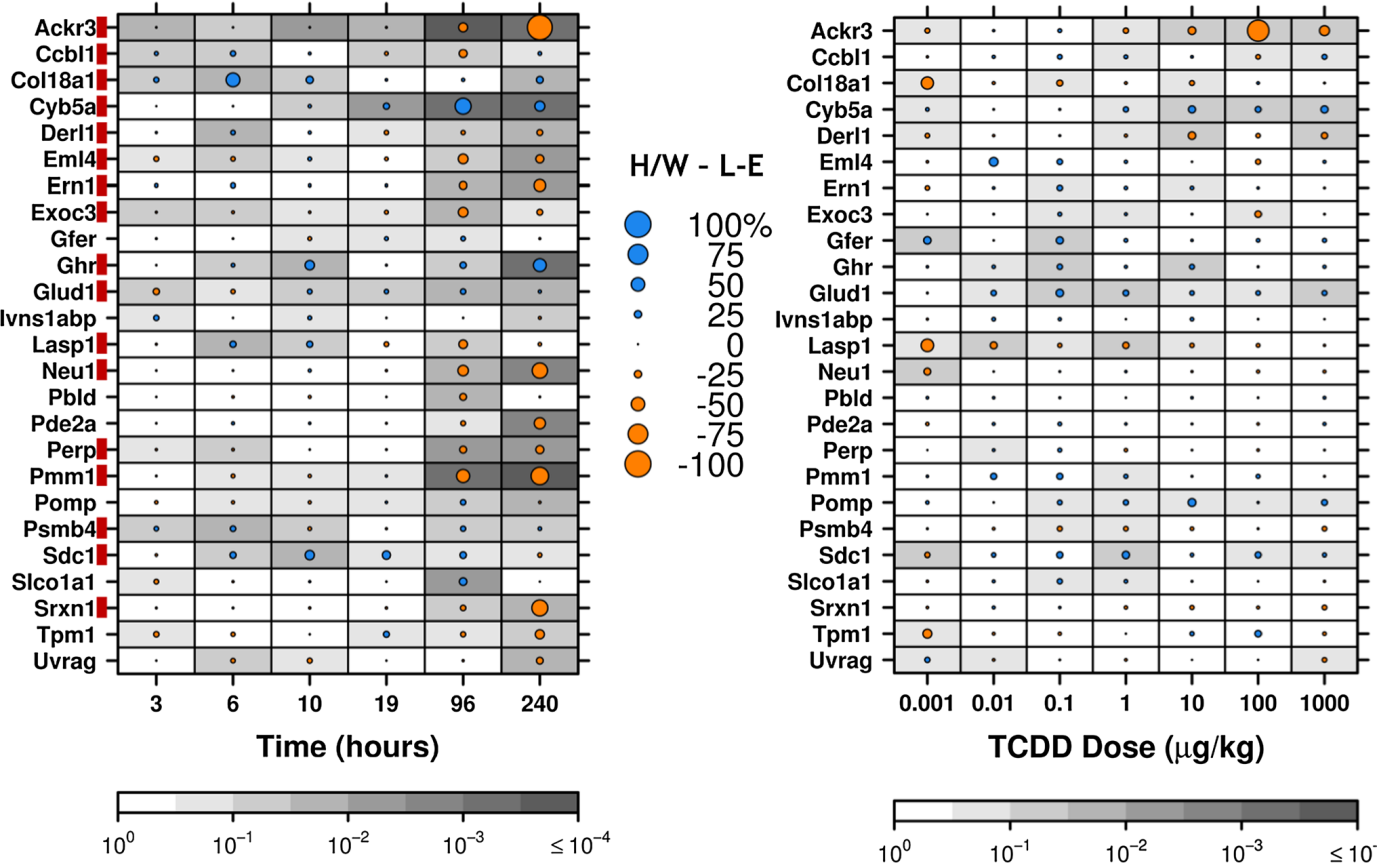
Summary of mRNA abundance changes following TCDD treatment. The dot size represents H/W per cent change—L–E per cent change values. Shading of individual squares represents the FDR-adjusted *p* value for an unpaired Student’s *t* test comparing the inter-strain differences. *Orange circles* indicate higher abundance in L–E, while blue circles indicate higher abundance in H/W. A *red box* to the right of the gene *symbol* indicates that this gene had a statistically significant difference between strains at two or more consecutive time points (colour figure online)

**Fig. 3:**
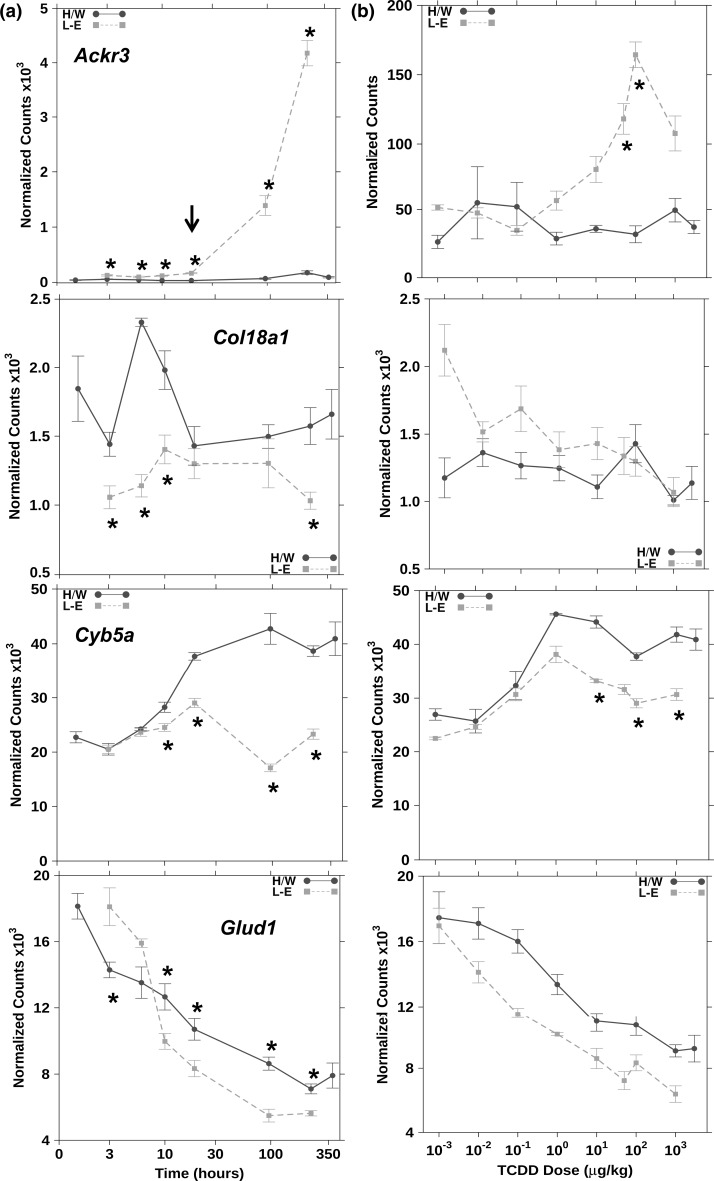
*Ackr3*, *Cyb5a*, *Col18a1 and Glud1* are genes with prolonged differential responses. Hepatic mRNA abundances of *Ackr3*, *Cyb5a*, *Col18a1* and *Glud1* display significantly differences in response between strains at three or more consecutive time points following TCDD treatment. a Normalized mRNA abundance time course profiles of TCDD-treated animals; b animals were similarly evaluated along a dose–response study with samples collected at 19-h post-treatment. *Asterisk* indicates *p*_adjusted_ < 0.1 when comparing H/W to L–E using an unpaired Student’s *t* test

The NanoString platform was used to analyse effects of TCDD treatment on the mRNA abundance of a subset of TCDD-regulated genes in livers of H/W and L/E rats. The utility of the approach has been validated previously by analysis of a well-characterized TCDD-regulated gene, *Cyp1a1* (Watson et al. 2013). A summary of the mRNA abundance changes for all genes examined is shown in Fig. 1. Eight genes showed similar mRNA responses following TCDD treatment in both strains (growth factor, augmenter of liver regeneration (*Gfer*), influenza virus NS1A-binding protein (*Ivns1abp*), phenazine biosynthesis-like protein domain containing 1 (*Pbld*), phosphodiesterase 2A (*Pde2a*), proteasome maturation protein (*Pomp*), solute carrier organic anion transporter family, member 1a1 (*Slco1a1*), tropomyosin 1, alpha (*Tpm1*) and UV radiation resistance-associated gene (*Uvrag*); Figs. S2–S9). Differences in the mRNA response profiles for L–E and H/W rats were defined as those with significantly different inter-strain mRNA abundances (in the same direction) at two or more consecutive time points (*p*_adjusted_ < 0.10). This criterion was met for 17/25 genes (*Ackr3*, cysteine conjugate-beta lyase, cytoplasmic (*Ccbl1*), *Col18a1*, *Cyb5a*, derlin 1 (*Derl1*), echinoderm microtubule-associated protein like 4 (*Eml4*), endoplasmic reticulum to nucleus signalling 1 (*Ern1*), exocyst complex component 3 (*Exoc3*), growth hormone receptor (*Ghr*), *Glud1*, LIM and SH3 protein 1 (*Lasp1*), neuraminidase 1 (*Neu1*), TP53 apoptosis effector (*Perp*), phosphomannomutase 1 (*Pmm1*), proteasome subunit beta 4 (*Psmb4*), syndecan 1 (*Sdc1*) and sulfiredoxin 1 (*Srxn1*); Fig. 2, Figs. S10–S22). Furthermore, we deemed it most likely that a gene responsible for prolonged toxicity would exhibit significant inter-strain differences at three or more consecutive time points. Using these criteria, four genes were identified as being potentially involved in L–E-specific hepatic toxicity (*Ackr3*, *Cyb5a*, *Col18a1* and *Glud1*, Fig. 3). While *Ghr* did not meet the above criteria for inter-strain differences, it demonstrated significantly different inter-strain mRNA abundance at four of the six time points (Fig. S15).

Genes could also be subdivided into groups defined by the time at which mRNA abundance began to deviate between H/W and L–E. Nine genes were observed to have differential inter-strain mRNA abundances beginning earlier than 10 h post-treatment (*Ackr3*, *Ccbl1*, *Col18a1*, *Exoc3*, *Ghr*, *Glud1*, *Lasp1*, *Psmb4* and *Sdc1*; Fig. 2 and Figs. S10, S14–S16, S20, S21), while eight genes deviated at 10 h or later after TCDD exposure (*Cyb5a*, *Derl1*, *Eml4*, *Ern1*, *Neu1*, *Perp*, *Pmm1* and *Srxn1*; Fig. 3 and Figs. S11–S13, S17–S19, S22).

### “Biphasic” responses

Nine genes demonstrated “biphasic” mRNA abundance changes in response to the 100 µg/kg TCDD treatment. These genes reached an initial plateau or peak early after treatment (between 3- and 10-h post-treatment) followed by a secondary response, which in most cases represented an extension or exaggeration of the original change. The exception to this trend was *Ern1*, where an initial repression caused by TCDD treatment was reversed beginning at 19-h post-treatment in L–E rat liver. The abundance of *Ern1* rapidly returned to near control levels in L–E animals, but remained reduced in H/W liver (Fig. S13). In all other instances (*Ackr3*, *Ccbl1*, *Exoc3*, *Neu1*, *Pde2a*, *Perp*, *Pmm1* and *Sxrn1*; Fig. 3; Figs. S5, S10, S14 S17–S19 and S22), L–E animals displayed a secondary exaggeration of the initial TCDD-induced change. H/W animals often exhibited the biphasic mRNA abundance pattern; however, with the exception of *Ern1*, the L–E secondary response occurred to a much larger magnitude than that observed in H/W liver.

### Dose–response analysis

Dose–response analyses were performed for all genes of interest at 19-h post-TCDD treatment. In most instances, the log dose–response curves presented the expected classic sigmoidal shape (Fig. S23). For some genes, both the dose–response and time course exhibited a muted response, indicating that the gene was poorly or non-responsive to TCDD at 19 h (*Uvrag*; Fig. 1; Figs. S9, S23). In general, the dose–response profiles showed less inter-strain variation than the time course analyses. For instance, *Ccbl1* displayed significant inter-strain differences in mRNA abundance at 3-, 6- and 240-h post-treatment; however, no difference was detected at the 19-h time point used for the dose–response study (Fig. S10). In contrast, *Cyb5a* is near its maximal time course response in H/W rats at 19 h and this was reflected by the changes observed using the dose–response approach (Fig. 3, Fig. S23). Only two genes had significant inter-strain differences in their ED_50_ values (*Cyb5a* and *Psmb4*; Table 1; Fig. S23). However, *Ackr3* could also be included here as it showed a very clear difference in the dose–response; L–E rats had an ED_50_ of 8.3 µg/kg, whereas the ED_50_ for H/W was not determinable since this gene was unresponsive in this strain until 240-h post-exposure (Fig. 3). Three additional genes had an ED_50_ determined for only one strain (*Lasp1*, *Srxn1* and *Tpm1*). *Lasp1* and *Srxn1* were determined to have an ED_50_ of 4.06 and 0.60 µg/kg, respectively, in L–E animals while *Tpm1* had an ED_50_ of 0.30 in H/W rats (Table 1 and Fig. S23).

**Table 1:** Genes analysed for differential responses

Gene symbol	TCDD response	Strain-related difference	ED_50_ H/W µg/kg	ED_50_ L–E µg/kg	Difference as per cent	Absolute difference	Gene ID
*Ackr3*	Induced	Higher in L–E	ND	8.30^a^	96.0	3984	84,348
*Ccbl1*	Induced	Transiently lower in L–E	3.93^a^	4.13^a^	28.3	460	311,844
*Col18a1*	Repressed	Lower in L–E	ND	ND	51.6	1204	85,251
*Cyb5a*	Induced (H/W)	Higher in H/W	0.10^a^	0.01^b^*	59.8	25,321	64,001
*Derl1*	Induced	Higher in L–E	7.43	1.06^a^	19.6	260	362,912
*Eml4*	Induced	Higher in L–E	0.32	0.94^a^	36.3	539	313,861
*Ern1*	Repressed	Higher in L–E	3.47	0.05	48.9	136	498,013
*Exoc3*	Induced	Higher in L–E	2.28^a^	4.42^a^	35.3	780	252,881
*Gfer*	None	None	ND	0.62	15.0	74	27,100
*Ghr*	Repressed	Lower in L–E	0.62	0.31	56.4	2303	25,235
*Glud1*	Repressed	Lower in L–E	3.07	1.22	36.7	1291	24,399
*Ivns1abp*	Repressed	None	1.65	1.55	16.4	192	289,089
*Lasp1*	Repressed	Variable	ND	4.06	30.0	195	29,278
*Neu1*	Induced	Higher in L–E	0.56	2.74	56.9	499	24,591
*Pbld*	Induced	None	0.47	0.56	12.3	2143	171,564
*Pde2a*	Induced	None	0.33	0.41	40.7	371	81,743
*Perp*	Induced	Higher in L–E	1.49	1.04	28.4	904	292,949
*Pmm1*	Induced	Higher in L–E	1.76	1.01	66.4	372	300,089
*Pomp*	None	None^c^	ND	ND	20.5	851	288,455
*Psmb4*	Repressed	Transiently lower in L–E	1.07	0.10*	22.9	394	58,854
*Sdc1*	Repressed	Transiently lower in L–E	0.46	0.27	38.7	513	25,216
*Slco1a1*	Repressed	None	5.84	0.05	35.6	261	50,572
*Srxn1*	Induced	Higher in L–E	ND	0.60	61.7	252	296,271
*Tpm1*	Induced	None	0.30	ND	38.0	26	24,851
*Uvrag*	None	None	ND	ND	24.1	117	308,846

The column labelled “Absolute Difference” denotes the maximal absolute difference in mRNA counts (time course) between L–E and H/W rats. The “Difference as Percent” column is the “Absolute Difference” value as a per cent of the maximal TCDD-induced change for that gene. *Indicates a difference with *p*_adjusted_ value <0.1 for between strain ED_50_ values

^a^ED_50_ value significantly different from the prototypical AHR-regulated gene, *Cyp1a1* (Watson et al. 2013)

^b^Significant equivalence to *Cyp1a1*

^c^ *Pomp* shows two consecutive points that are significantly different between strains; however, the differences are not consistent—one time point has significantly reduced abundance, whereas the other shows significant induction. ND indicates that the ED50 could not be determined for that gene

### Sensitivity to TCDD

Of the 25 genes we examined, only *Cyb5a* (L–E, ED_50_ 0.01) demonstrated TCDD sensitivity equivalent to the prototypic AHR-regulated gene, *Cyp1a1* [ED_50_ 0.013 (H/W), 0.035 (L–E), (Watson et al. 2013)], while six genes displayed lower sensitivity. These genes [*Ackr3*, *Ccbl1* (L–E), *Cyb5a* (H/W), *Derl1* (L–E), *Eml4* (L–E) and *Exoc3*; Table 1, Fig. 23] had an ED_50_ significantly higher than that of *Cyp1a1*. Of these, *Ackr3* was the only gene with an ED_50_ similar to the LD_50_ of male L–E rats (8.62 vs. 17.7 µg/kg, respectively), while having an undetermined ED_50_ in H/W.

## Discussion

Previously, we identified 30 genes that exhibited concordant hepatic mRNA responses between two TCDD-sensitive rodent species following TCDD treatment, along with three genes that demonstrated divergent responses (Boutros et al. 2008). These 33 genes are candidate mediators of TCDD-induced hepatotoxicity in TCDD-sensitive rodents. Liver was selected for study because numerous studies show extensive biochemical and pathologic changes in liver following dioxin exposure (Forgacs et al. 2012; Pohjanvirta et al. 1989, 1990; Viluksela et al. 2000, 1999). Further, unlike other potential target organs such as white adipose tissue or hypothalamus where few mRNAs are altered by TCDD exposure (Houlahan et al. 2015a, b), hundreds to thousands of rat liver genes are modulated by the activated AHR following TCDD exposure (Boutros et al. 2011; Boverhof et al. 2006; Fletcher et al. 2005; Franc et al. 2008; Vezina et al. 2004; Yao et al. 2012). Our goal was to prioritize the 25 non-“AHR-core” genes of this cohort for further mechanistic investigation. Rat strains with striking differences in susceptibility to TCDD toxicities were selected: H/W rats are essentially unaffected by doses that are lethal to L–E rats (Tuomisto et al. 1999). Inter-strain differences in the abundance profiles for a specific mRNA that occur before or at the onset of toxicity may indicate genes mechanistically involved in TCDD-induced type II toxicity. Further, genes involved in L–E-specific toxicity might be expected to be more sensitive to TCDD treatment, having a lower ED_50_ for these genes in L–E than in H/W animals, or the genes may only be responsive in L–E. It has been shown that the earliest manifestations of toxicity occur rapidly, with TCDD-induced weight loss and changes in blood chemistry measurable within 24 h (Linden et al. 2014). Interestingly, the onset of biochemical or physiological changes in response to TCDD occurs at a time very close to that observed for the “biphasic” changes in mRNA abundance suggested for *Ackr3*, *Ccbl1*, *Ern1*, *Exoc3*, *Neu1*, *Pde2a*, *Perp*, *Pmm1* and *Sxrn1*.

Of the 25 genes examined, eight are unlikely to be directly involved in type II toxic responses to TCDD, since they exhibited similar responses to TCDD in both TCDD-sensitive and TCDD-resistant strains throughout the time course study and at all doses tested (*Gfer*, *Ivns1abp*, *Pbld*, *Pde2a*, *Pomp*, *Slco1a1*, *Tpm1* and *Uvrag* Fig. S2–S9). The remaining 17 displayed some degree of inter-strain differential mRNA abundance following TCDD exposure. Most of these demonstrated enhanced or exaggerated effects in response to TCDD exposure in TCDD-sensitive L–E rats. Only *Cyb5a* (one of the four genes that displayed a significant, prolonged inter-strain difference) had an enhanced response in the TCDD-resistant H/W liver, with an ~twofold up-regulation beginning early (6 to 10 h) after exposure, as compared to essentially no change in L–E liver (Fig. 3). This H/W-specific gene modulation had previously been observed for *Cyb5a* and other six genes (Boutros et al. 2011). It is possible that *Cyb5a* and other H/W-specific gene responses to TCDD play a protective role, ameliorating toxic outcomes. Comparison of the genomic DNA sequences for H/W and L–E rat did not identify any differences in AHREs within 3 kilobases of the transcriptional start site for any of the genes (Boutros, PC and Prokopec SD, in preparation). Of note, *Cyb5a* has recently been shown to be involved in the kynurenine pathway, its gene product acting as the major reducing agent of indoleamine 2,3-dioxygenase (IDO), the first and rate-limiting step (Maghzal et al. 2008). Altered tryptophan metabolism following TCDD treatment with increased circulating levels of tryptophan in TCDD-sensitive rat strains including L–E and concomitant decreases in tryptophan dioxygenase activity in rat liver has been observed (Unkila et al. 1994, 1995, 1998, 1999; Weber et al. 1994). Further, *Cyb5a* has been shown to play a role in promoting autophagy in pancreatic cancer cells (Giovannetti et al. 2014). Promotion of autophagy has also been shown to reduce steatohepatitis and fibrosis in mouse liver (Lodder et al. 2015; Zhong et al. 2015), perhaps representing a mechanism by which *Cyb5a* protects H/W rats.

Following TCDD treatment, liver *Ghr* (growth hormone receptor) is lower at four time points, separated by a single non-significant difference at 19 h in TCDD-sensitive L–E when compared to that observed in TCDD-resistant H/W (Fig. S15). This gene could be involved in both early and late responses to TCDD exposure. AHR activation leads to suppression of *Ghr* mRNA levels in livers of TCDD-sensitive mice (Nukaya et al. 2004). Reduced *Ghr* mRNA abundance in TCDD-sensitive L–E rats may play a significant role in the pathogenesis of many well-known TCDD-induced toxic outcomes. Following a lethal dose of TCDD, L–E rat liver undergoes accumulation of fat and infiltration of inflammatory cells (steatohepatitis), while this does not occur in H/W rats given the same dose of TCDD (Pohjanvirta et al. 1989, 1990). Similarly, reduction in growth hormone signalling by liver-specific knockout of *Stat5* leads to steatohepatitis, glucose intolerance, late onset obesity, impaired liver regeneration and insulin resistance (Baik et al. 2011). Liver-specific knockout of *Ghr* in mice recapitulated the *Stat5* knockout phenotype and also led to non-alcoholic fatty liver disease, fibrosis and hepatocellular carcinoma (Fan et al. 2014). Signalling through the GHR also directly affects metabolism and insulin secretion (Strobl and Thomas 1994), as well as sex steroid metabolism (Baik et al. 2011), immune function and apoptosis (Savino et al. 2002).

The remaining three genes with prolonged inter-strain differences produce proteins involved in metabolic processes, angiogenesis, cytokine response, liver survival, liver repair and regeneration. The first of these, *Glud1* (glutamate dehydrogenase 1), is a mitochondrial enzyme that catalyses the reversible conversion of glutamate to α-ketoglutarate and regulates several important metabolic and neurological pathways. The mRNA abundance of *Glud1* is reduced in both strains but to a greater extent in TCDD-sensitive L–E rats. Glutamate plays a key role in regulation of energy homoeostasis in an organ-specific manner (reviewed by Karaca et al. 2011). In pancreatic islet cells for instance, decreased *Glud1* activity reduced insulin release, leading to organism-wide metabolic alterations. Reduced plasma insulin levels following TCDD treatment in Sprague–Dawley rats have been observed (Gorski et al. 1988; Gorski and Rozman 1987).

*Col18a1* mRNA abundance was significantly lower in L–E rat at early (3–10 h, Fig. S3) and late time points following TCDD insult. It will be interesting to determine whether the decreased mRNA abundance is correlated with decreased amounts of both mature COL18A1 and/or decreased amounts of active peptide domains. These early differences between L–E and H/W may indicate that *Col18a1* is involved in the early stages of TCDD hepatotoxicity, while the late difference may indicate it also is involved in TCDD-induced cancer or other delayed toxicities (Viluksela et al. 2000). COL18A1 mutations that lead to deficiency in its cleavage product, endostatin, have been shown to lead to cancer (Mahajan et al. 2010). Interestingly, COL18A1 contains amino terminal domains which, upon proteolytic cleavage, inhibit blood vessel formation (Zhuo et al. 2011), reduce cellular proliferation (Zhang et al. 2012) and block WNT signalling (Lavergne et al. 2011; Quelard et al. 2008; Seppinen and Pihlajaniemi 2011). Importantly, COL18A1 is an essential survival factor following acute liver toxicity from CCl_4_ (Duncan et al. 2013).

*Ackr3* displays the largest and most prolonged change that we observed in L–E rat (Fig. 3, all time points and maximally >16-fold difference from H/W rat). Interestingly, the ED_50_ for *Ackr3* in L–E rats is ~8.3, while the there was no change observed in H/W rat for any doses tested at 19 h. Since *Ackr3* responds only in the sensitive L–E strain, has an early response and exhibits an ED_50_, similar to the LD_50_ for TCDD in L–E rats (male ~17.7 µg/kg), it closely resembles the expected profile for genes causative of TCDD toxicity. It has been shown that the ED_50_ values for toxic outcomes following TCDD exposure, such as thymic atrophy and wasting syndrome, are similar to the LD_50_ values in Sprague–Dawley rats (Hanberg et al. 1989). Ackr3 binds to cytokines SDF-1 and ITAC, and has been implicated in cellular migration and invasion (Naumann et al. 2010; Tarnowski et al. 2010). *Ackr3* has also been implicated in hypoxia response, tumour development, cell growth, cell survival and adhesion (Burns et al. 2006; Hu et al. 2010; Liu et al. 2010; Staton et al. 2011; Sun et al. 2010). Further, it plays a role in the brain and may be involved in modulation of anxiety and other behaviour (Guyon 2014; Ikeda et al. 2013). Recently, *Ackr3* has been identified as a liver injury-inducible liver sinusoidal endothelial cell (LSEC)-specific SDF-1 receptor (Ding et al. 2014). Induction of *Ackr3* in LSECs stimulates liver regeneration and reduces fibrosis. This is unexpected since other reports have shown that TCDD exposure increases expression of molecular markers of fibrosis in mice (Pierre et al. 2014). Activated *Ackr3* has been shown to increase uptake of VLDL and cholesterol into adipose tissue, reducing circulating levels (Li et al. 2014). It is expressed at very low levels in normal hepatic tissue, but is highly expressed in murine hepatocellular carcinoma, predominantly in epithelial cells (Monnier et al. 2011). *Ackr3* inhibition or inactivation reduces head and neck tumour growth and increases survival of mice with brain cancer (Maussang et al. 2013; Walters et al. 2014).

Of the 33 genes identified in our previous comparison of two TCDD-sensitive rodent species, mice and rats, we analysed here the 25 non-“AHR-core” genes in-depth to further characterize candidate mediators of TCDD toxicity. Of these, four genes displayed an inter-strain difference that persisted for 240 h or more (*Ackr3*, *Cyb5a*, *Col18a1* and *Glud1*) with significantly different mRNA responses in livers of TCDD-resistant H/W versus TCDD-sensitive L–E rats. Since L–E rats are susceptible to TCDD-induced toxicities, whereas H/W rats are essentially refractory to them; these genes may play essential roles in the onset of toxicity. This study takes a key step towards identification of the specific genes and metabolic pathways which underlie toxic outcomes induced by TCDD by showing that eight TCDD-altered genes are unlikely to be involved in TCDD toxicity (*Gfer*, *Ivns1abp*, *Pbld*, *Pde2a*, *Pomp*, *Slco1a1*, *Tpm1* and *Uvrag*), while identifying four genes (*Ackr3*, *Cyb5a*, *Col18a1* and *Glud1*) that could play a key role in toxic outcomes. Future studies will be required to determine whether the reported changes in mRNA abundance lead to downstream changes in protein abundance, enzyme activities or sub-cellular location.

## Electronic supplementary material

Below is the link to the electronic supplementary material.

Supplementary material 1 (XLSX 18 kb)

Supplementary material 2 (PDF 1091 kb)

Supplementary material 3 (XLSX 775 kb)

Supplementary material 4 (TXT 11 kb)

Supplementary material 5 (TXT 10 kb)

Supplementary material 6 (TXT 27 kb)

Supplementary material 7 (TXT 45 kb)

Supplementary material 8 (FILE 6 kb)

Supplementary material 9 (TXT 4 kb)

Supplementary material 10 (XLSX 17 kb)

Supplementary material 11 (XLS 635 kb)

Supplementary material 12 (PDF 4277 kb)
