# Supplementary material for: 2,3,7,8 Tetrachlorodibenzo-p-dioxin-induced RNA abundance changes identify Ackr3, Col18a1, Cyb5a and Glud1 as candidate mediators of toxicity
Source: Arch Toxicol. 2016 Apr 30;91(1):325–38. doi: 10.1007/s00204-016-1720-0 (PMC5225275; doi:10.1007/s00204-016-1720-0)
Supplement: Supplementary file 12 — Supplementary material 12 (PDF 4277 kb) [file 204_2016_1720_MOESM12_ESM.pdf]

TCDD-induced RNA abundance changes identify candidate mediators of toxicity

John D. Watson[*], Stephenie D. Prokopec[*], Ashley B. Smith[*], Allan B. Okey[†], Raimo Pohjanvirta[‡,§], and Paul C. Boutros[*,¶]
[*]Informatics and Bio-computing Program, Ontario Institute for Cancer Research, Toronto, Canada.
[†]Department of Pharmacology and Toxicology, University of Toronto, Toronto, Canada.
[‡]Laboratory of Toxicology, National Institute for Health and Welfare, Kuopio Finland.
[§]Department of Food Hygiene and Environmental Health, University of Helsinki, Helsinki, Finland.
[¶]Department of Medical Biophysics, University of Toronto, Toronto, Canada.

Address for correspondence:

Dr. Paul C. Boutros

MaRS Centre, South Tower

101 College Street

Toronto, Ontario, Canada

M5G 0A3

Email: Paul.Boutros@oicr.on.ca
Phone: 416-673-8564
Fax: 416-673-8564

Supplementary information

Fig. S1 Schematic outline of experimental design. The times post-treatment (hours) at which livers were harvested are indicated below the horizontal arrow. TCDD dosages are indicated above the horizontal arrow (µg/kg), with dose-response treatments adjacent to the vertical arrow at the 19-hour time point. Dosages with a superscript are given only to one of the strains to adjust for their differing TCDD-sensitivity range. For example, 0.05L indicates that only L-E animals received this TCDD dose. Vehicle control times are indicated by the presence of a lower case l or h. For instance, there was a 1.5 hour H/W vehicle control (indicated by h above the horizontal line), and a 19-hour vehicle control for both H/W and L-E (indicated by lh).

Fig. S2 – S22 For consistency, the data for all genes are presented in 2 panels. Panel a) is time course data, while panel b) gives dose-response data. The graphs display normalized expression levels, providing a count of the gene-specific transcript in 100 ng of liver RNA. H/W data are represented by circles and L-E data are represented by squares. * indicates p-adj< 0.1 when comparing H/W to L-E using an unpaired Student's t-test. The abundance data shown are; Fig. 2 Gfer, Fig. 3 Ivns1abp, Fig. 4 Pbld, Fig. 5 Pde2a, Fig. 6 Pomp, Fig. 7 Slco1a1, Fig. 8 Tpm1, Fig. 9 Uvrag, Fig. 10 Ccbl1, Fig. 11 Derl1, Fig. 12 Eml4, Fig. 13 Ern1, Fig. 14 Exoc3, Fig. 15 Ghr, Fig. 16 Lasp1, Fig. 17 Neu1, Fig. 18 Perp, Fig. 19 Pmm1, Fig. 20 Psmb4, Fig. 21 Sdc1 and Fig. 22 Srxn1. The presence of an arrow indicates the proposed inflection point.

Fig. S23 A 4-parameter logistic model was used to determine ED50 values from dose-response data. Logistic fitting for each gene is shown, including ED50 values.

Fig. S24 Basal levels for genes analyzed in this study. Plots display mRNA counts from corn-oil gavage treated animals. Statistically significant inter-strain differences are indicated by asterisks ($p < 0.1$, Student's t-test with FDR correction).

File S1 Animal weights

File S2 Completed NC3Rs ARRIVE checklist

File S3 NanoString Codeset

File S4 Time course strain difference data and statistics

File S5 Dose-response strain difference data and statistics

File S6 NanoString time course statistics

File S7 NanoString dose-response statistics

File S8 Dose-response logistic fit data

File S9 Comparison and statistics for TCDD sensitivity vs Cyp1a1

File S10 Mean and standard deviation for ED50 values with equivalence testing vs Cyp1a1

File S11 Raw NanoString data with animal names and treatment.

Fig. S1 Experimental Design

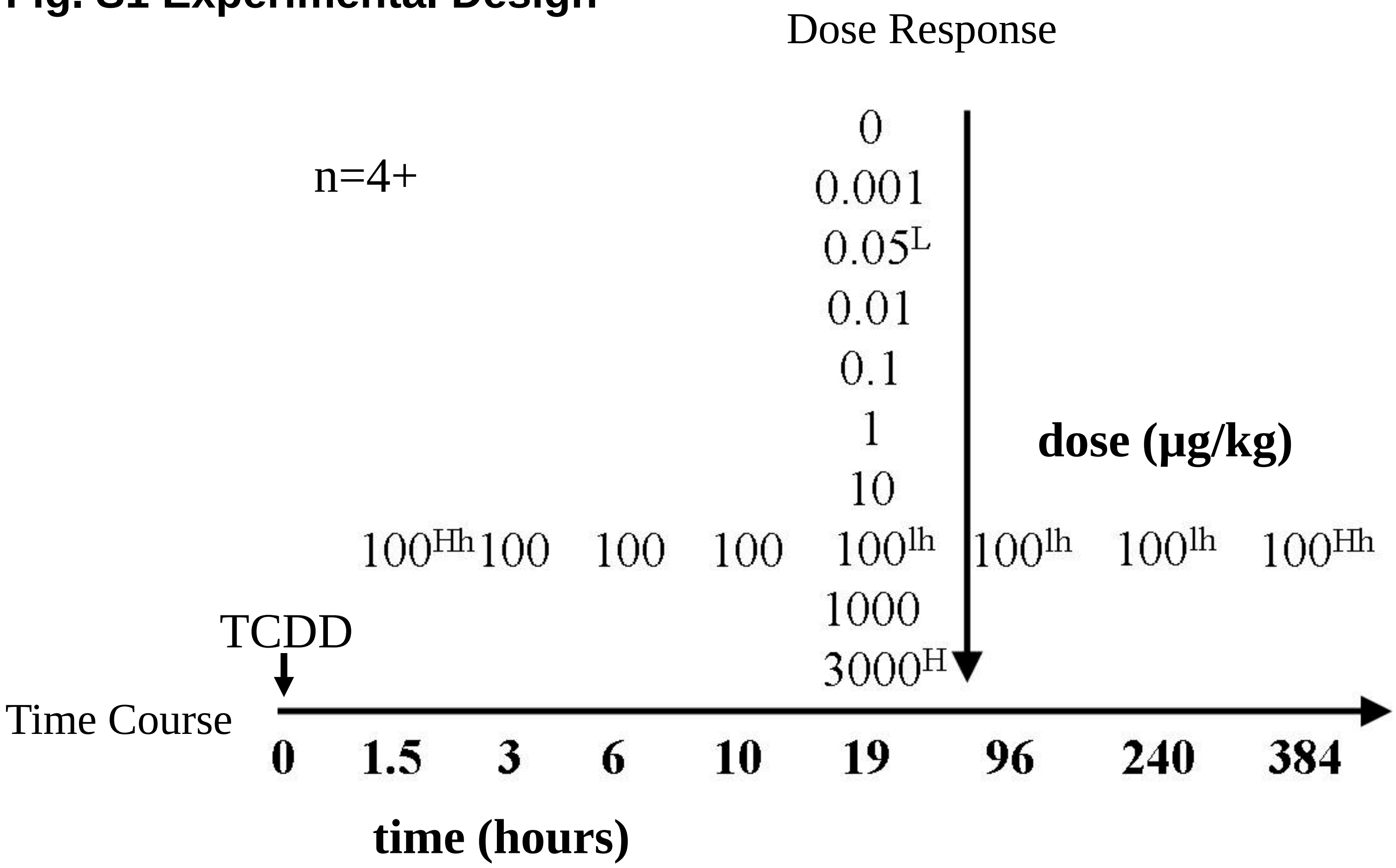

Fig. S2 *Gfer*

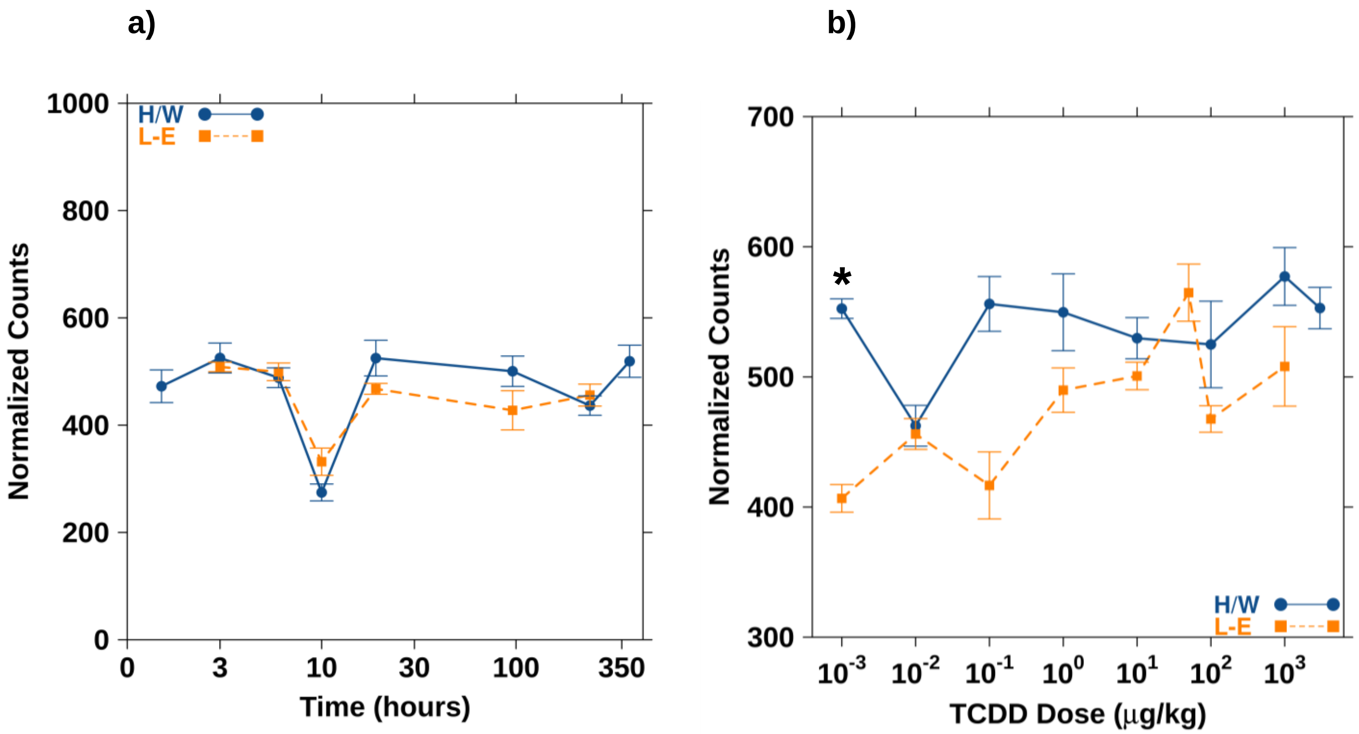
a)
b)
H/W
L-E
Normalized Counts
1000
800
600
400
200
0
0
3
10
30
100
350
Time (hours)
700
600
500
400
300
*
10^{-3} 10^{-2} 10^{-1} 10^{0} 10^{1} 10^{2} 10^{3}
TCDD Dose (μg/kg)

Fig. S3 *Ivns1abp*

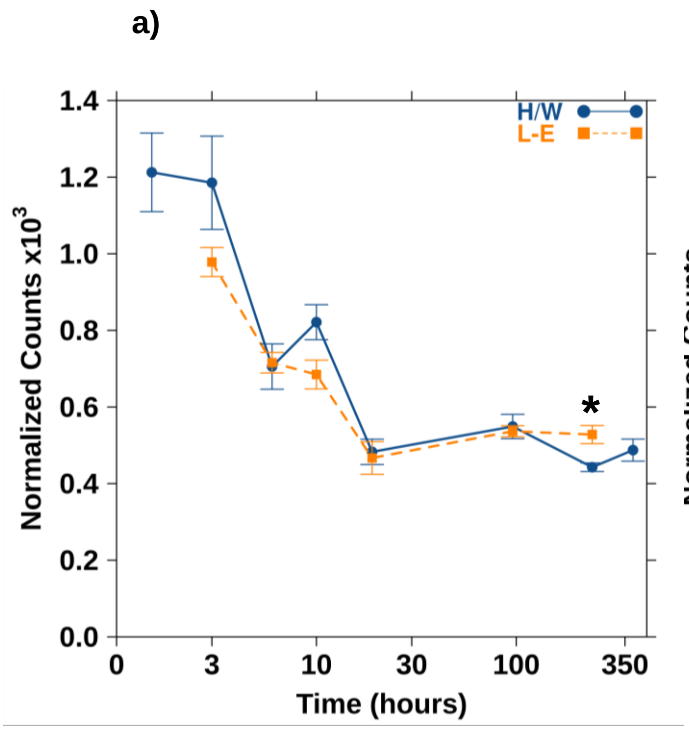

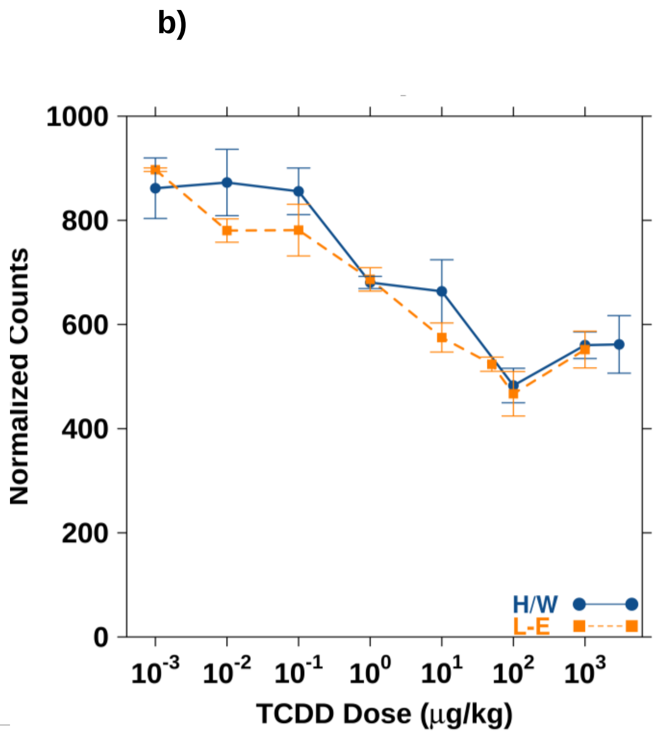

Fig. S4 *Pbld*

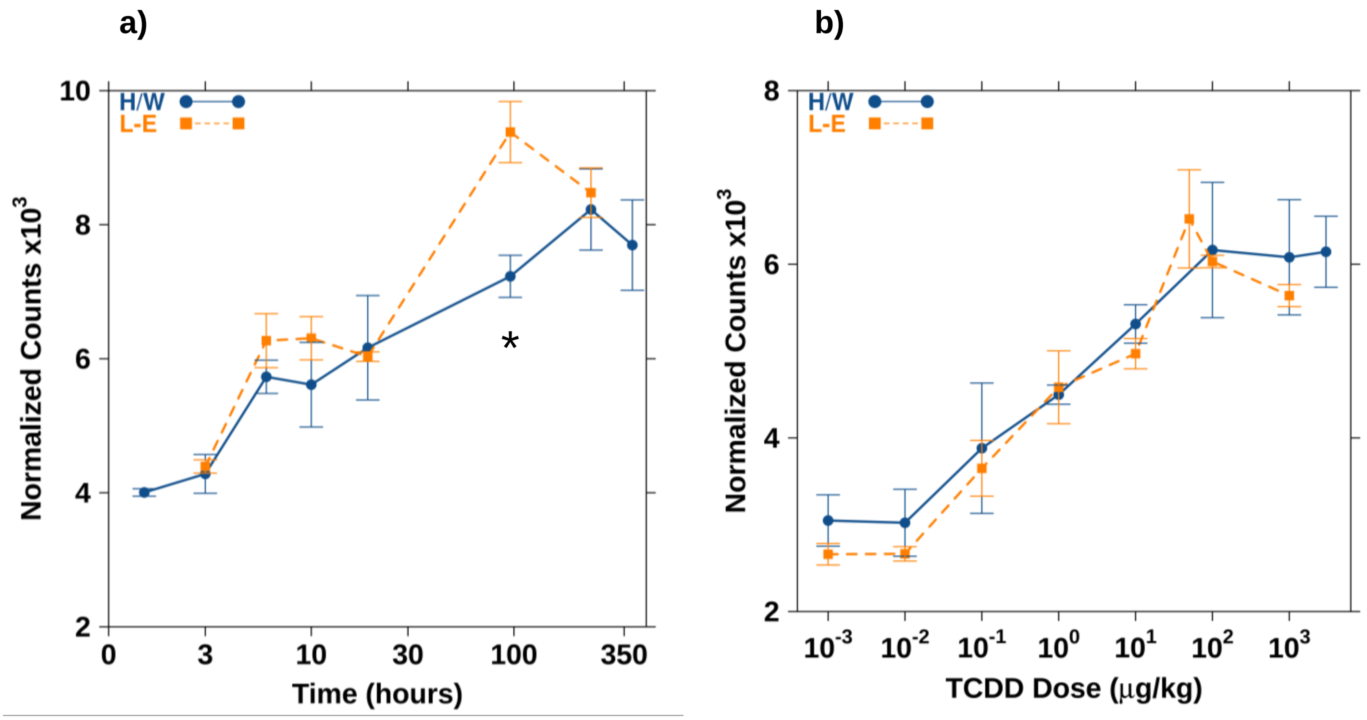

a)
H/W
L-E
Normalized Counts x10^3
10
8
6
4
2
0
3
10
30
100
350
Time (hours)
*
b)
H/W
L-E
Normalized Counts x10^3
8
6
4
2
10^-3
10^-2
10^-1
10^0
10^1
10^2
10^3
TCDD Dose (μg/kg)

Fig. S5 *Pde2a*

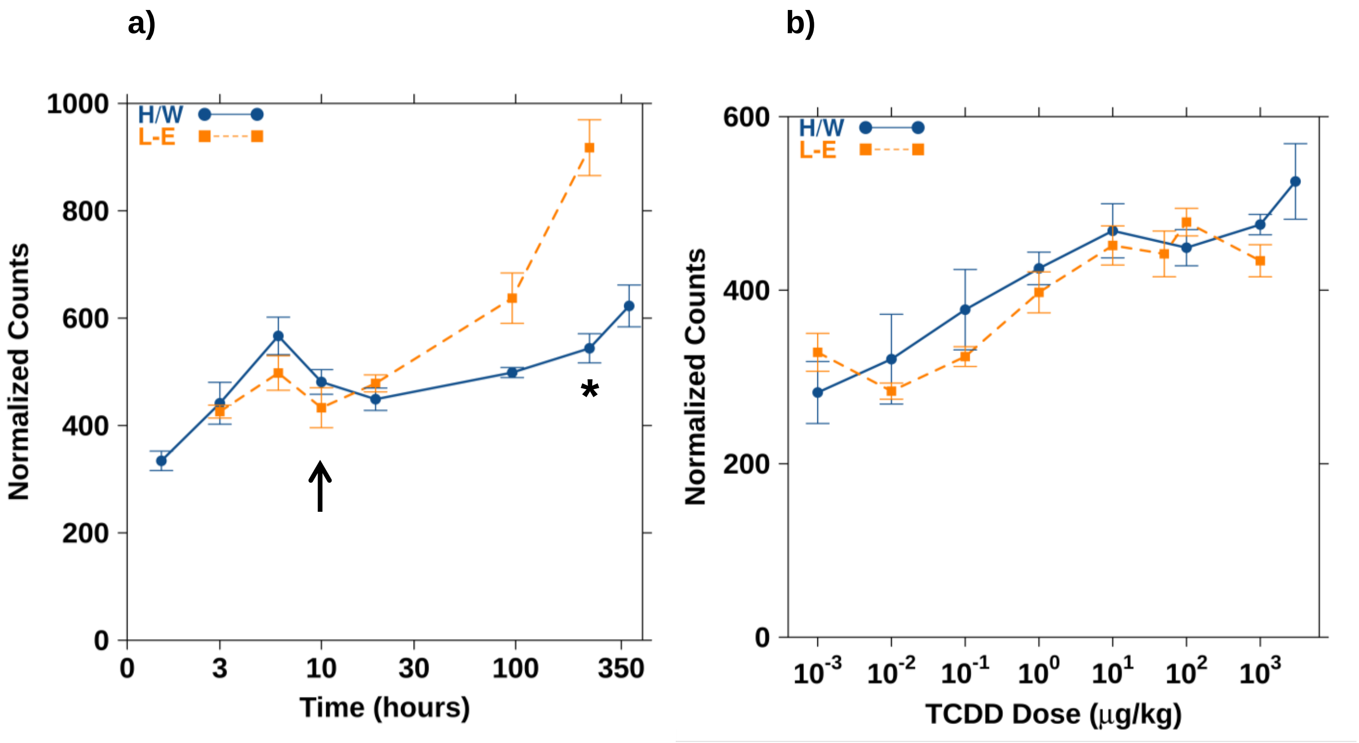
a)
b)
H/W
L-E
Normalized Counts
1000
800
600
400
200
0
3
10
30
100
350
Time (hours)
*
Normalized Counts
600
400
200
0
10^{-3}
10^{-2}
10^{-1}
10^{0}
10^{1}
10^{2}
10^{3}
TCDD Dose (μg/kg)

Fig. S6 *Pomp*

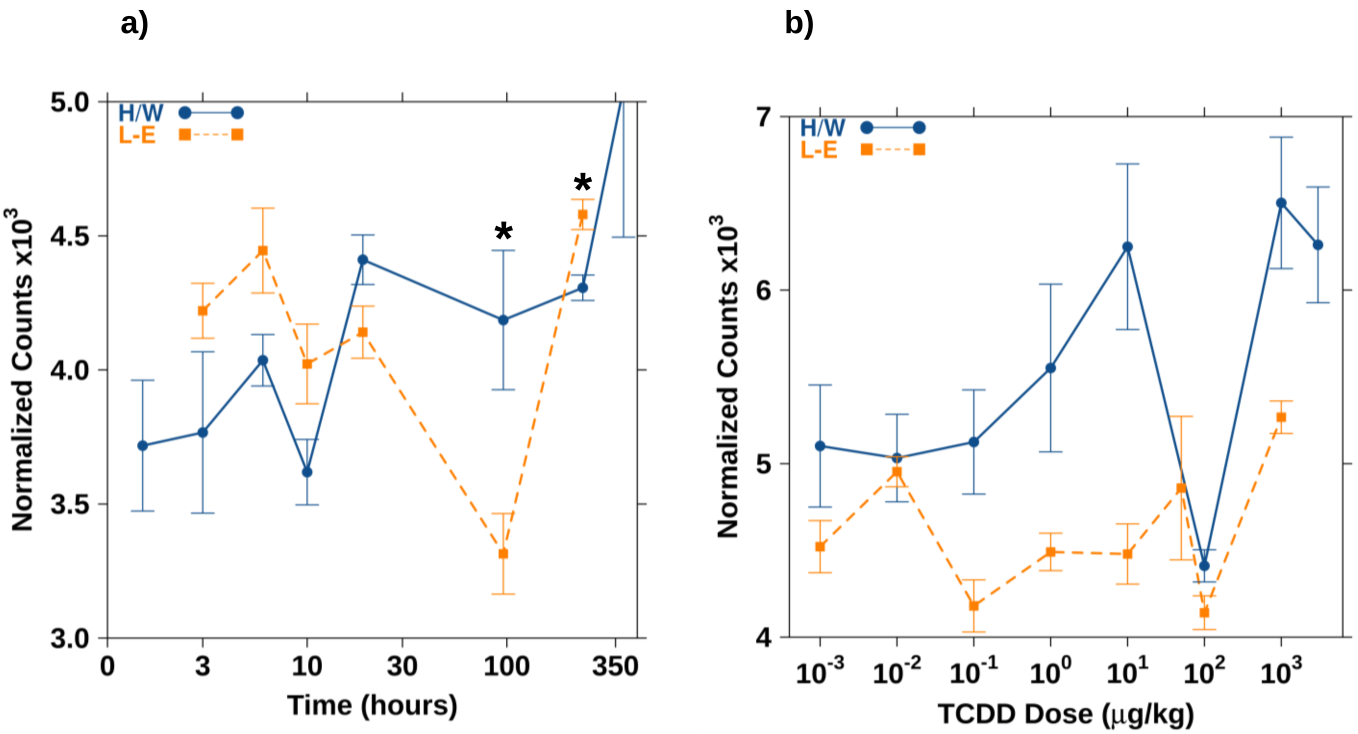
a)
b)
H/W
L-E
5.0
4.5
4.0
3.5
3.0
Normalized Counts x10^3
0
3
10
30
100
350
Time (hours)
*
*
7
6
5
4
10^-3
10^-2
10^-1
10^0
10^1
10^2
10^3
TCDD Dose (μg/kg)

Fig. S7 *Slco1a1*

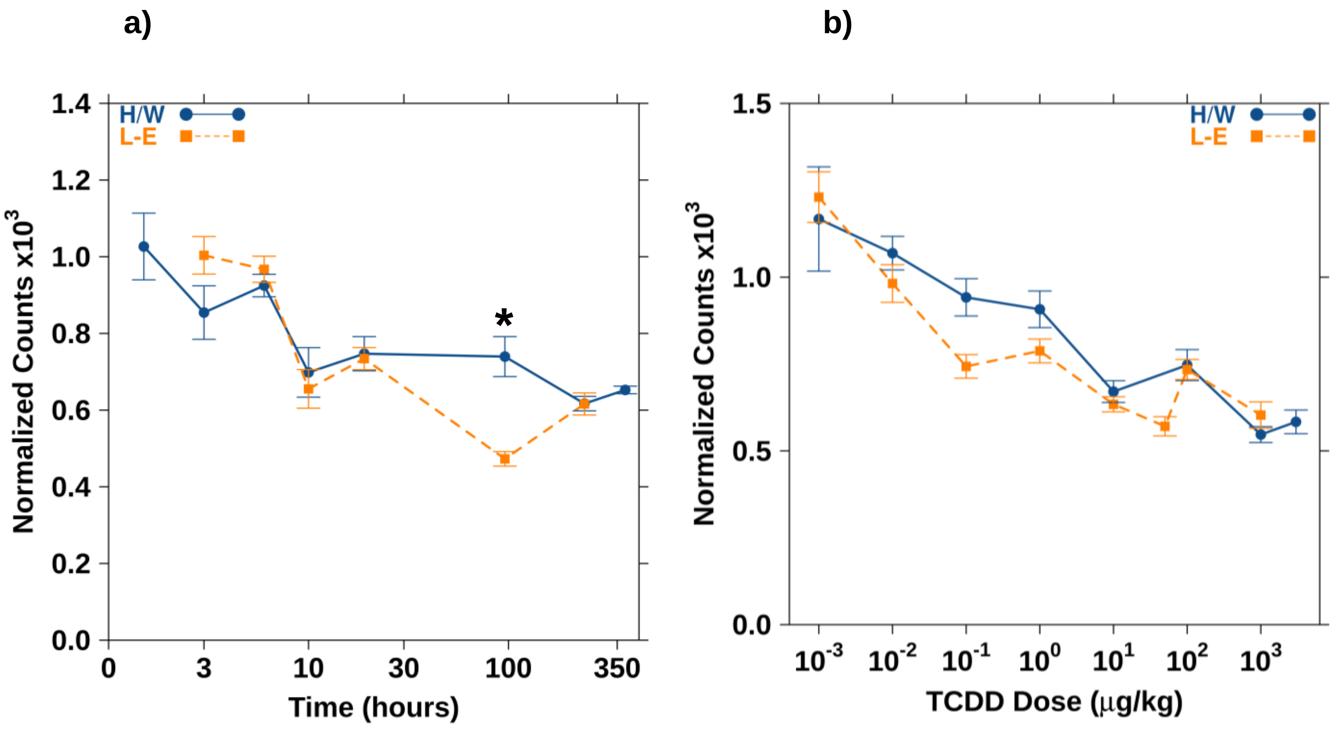

Fig. S8 *Tpm1*

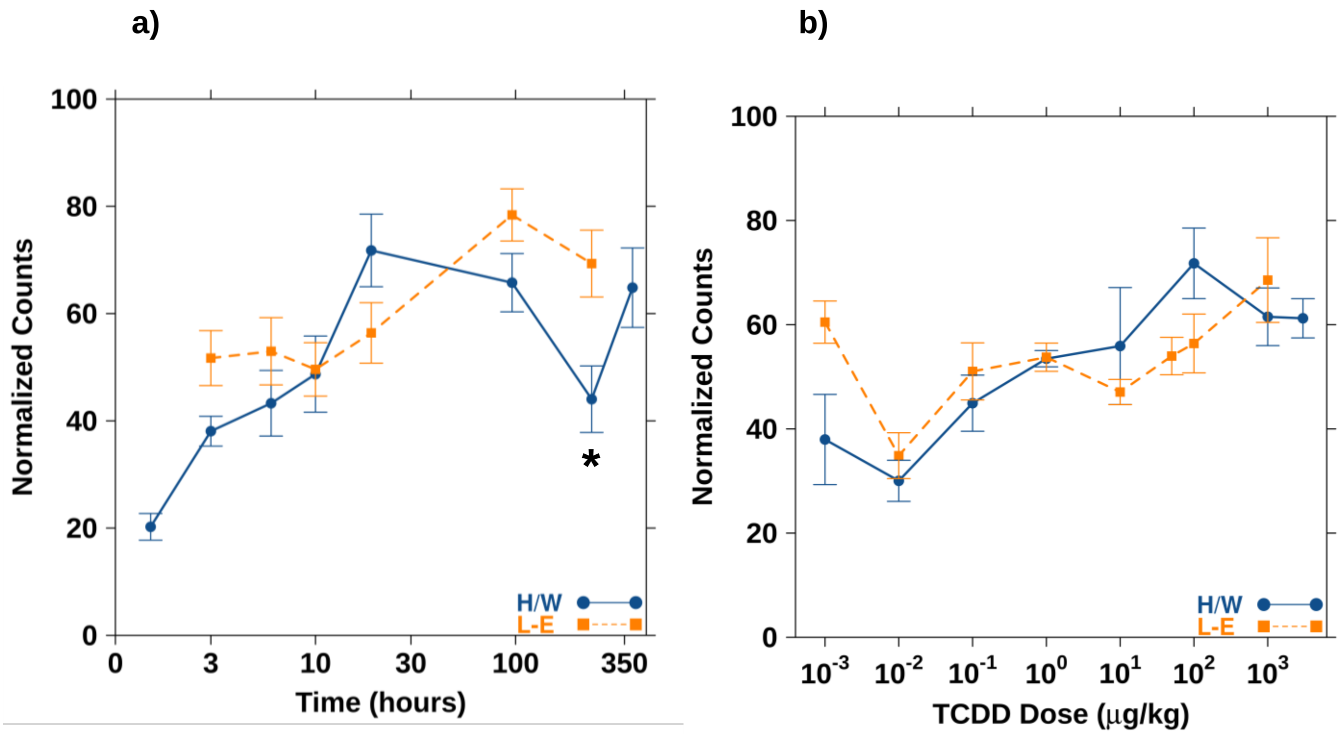

a)
b)
Normalized Counts
Time (hours)
0
3
10
30
100
350
0
20
40
60
80
100
*
H/W
L-E
TCDD Dose (μg/kg)
10-3
10-2
10-1
100
101
102
103

Fig. S9 *Uvrag*

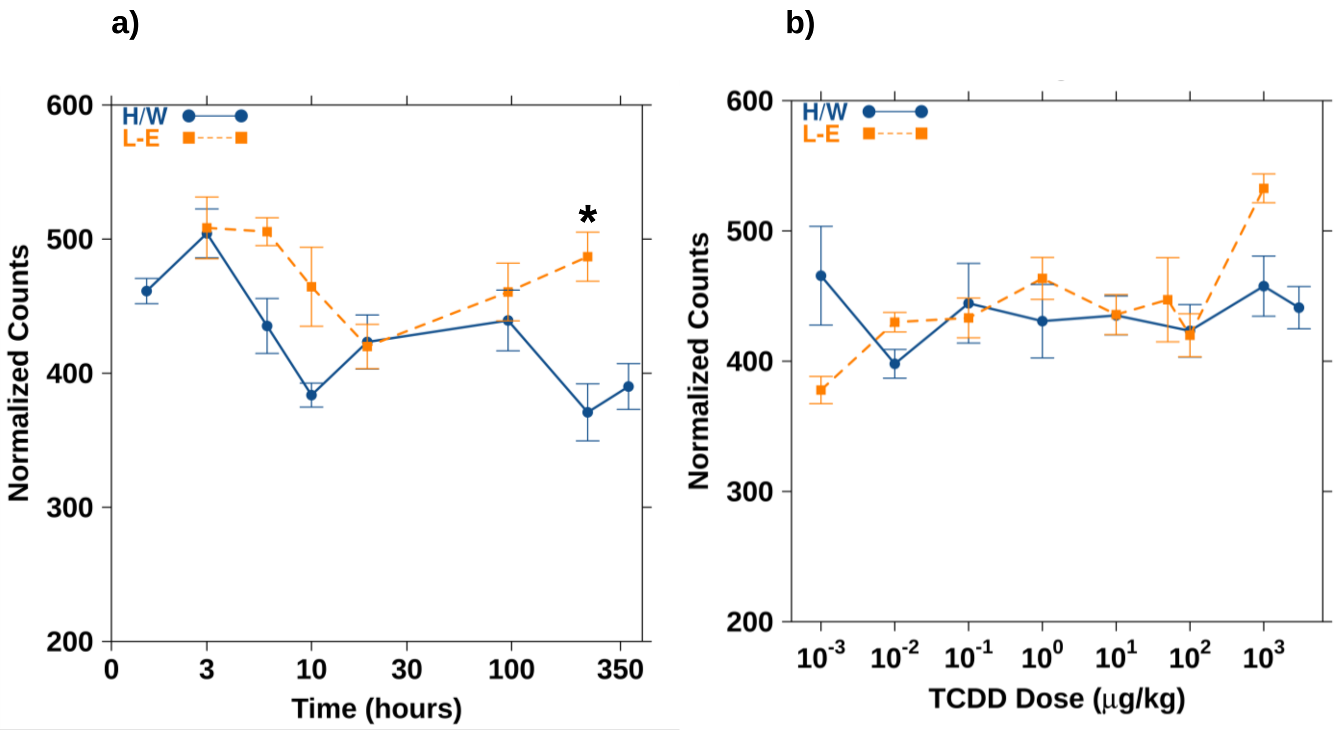
a)
b)
H/W
L-E
*
Normalized Counts
600
500
400
300
200
0
3
10
30
100
350
Time (hours)
10-3
10-2
10-1
100
101
102
103
TCDD Dose (μg/kg)

Fig. S10 *Ccbl1*

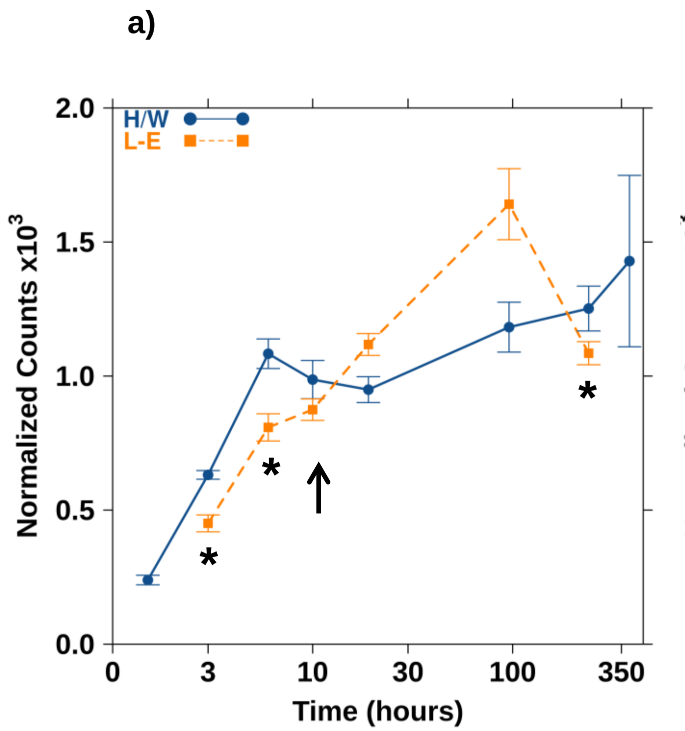

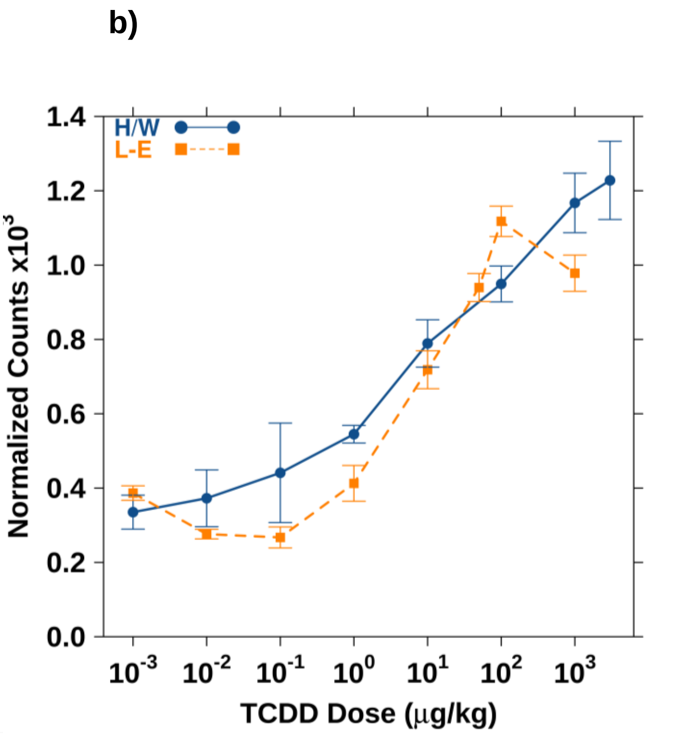

Fig. S11 *Derl1*

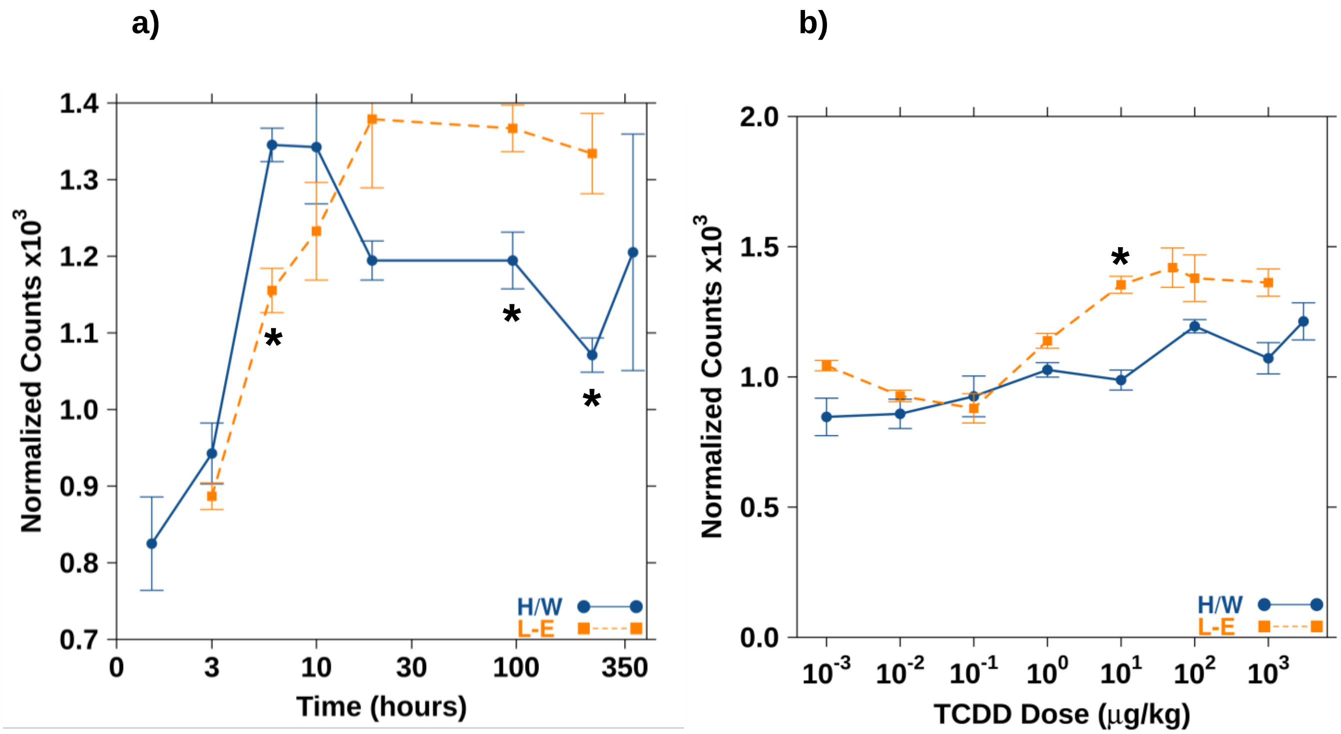

Fig. S12 *Eml4*

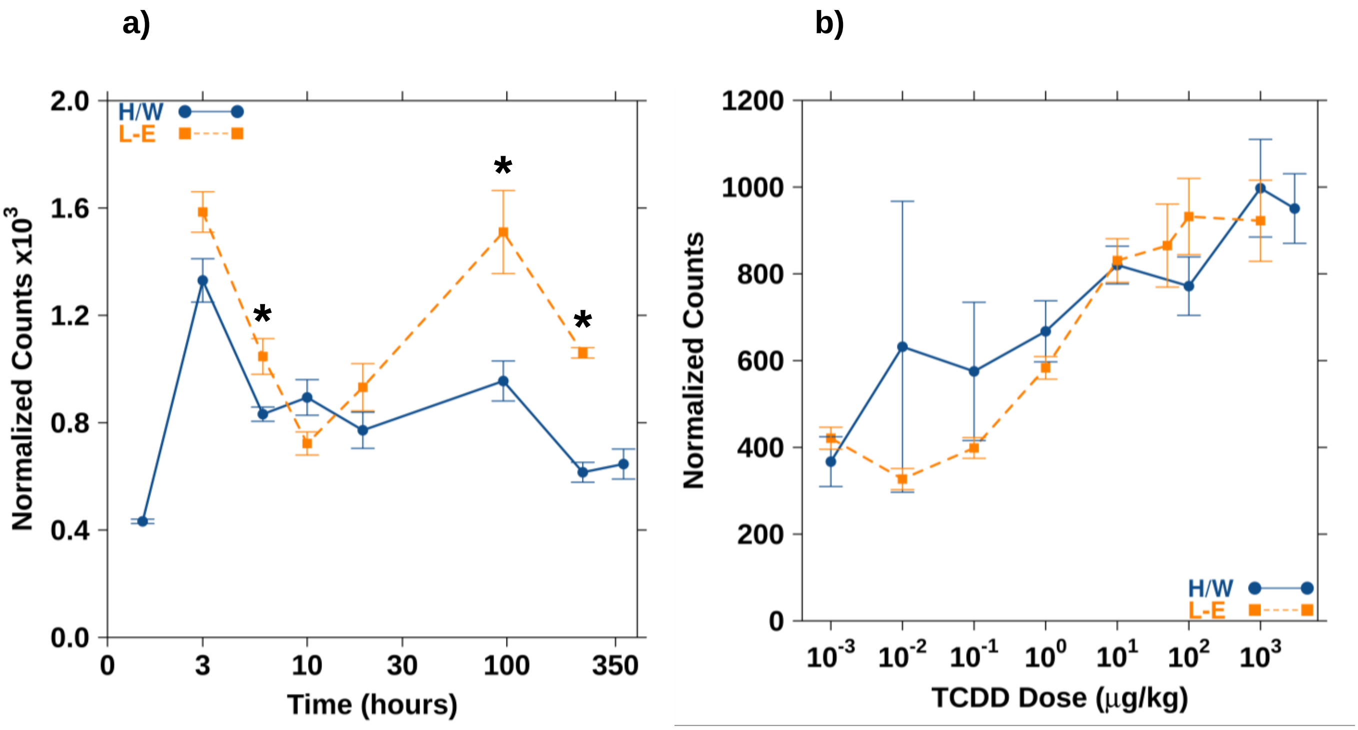
a)
b)
H/W
L-E
Normalized Counts x10^3
2.0
1.6
1.2
0.8
0.4
0.0
0
3
10
30
100
350
Time (hours)
*
*
*
Normalized Counts
1200
1000
800
600
400
200
0
10^{-3}
10^{-2}
10^{-1}
10^{0}
10^{1}
10^{2}
10^{3}
TCDD Dose (μg/kg)
H/W
L-E

Fig. S13 *Ern1*

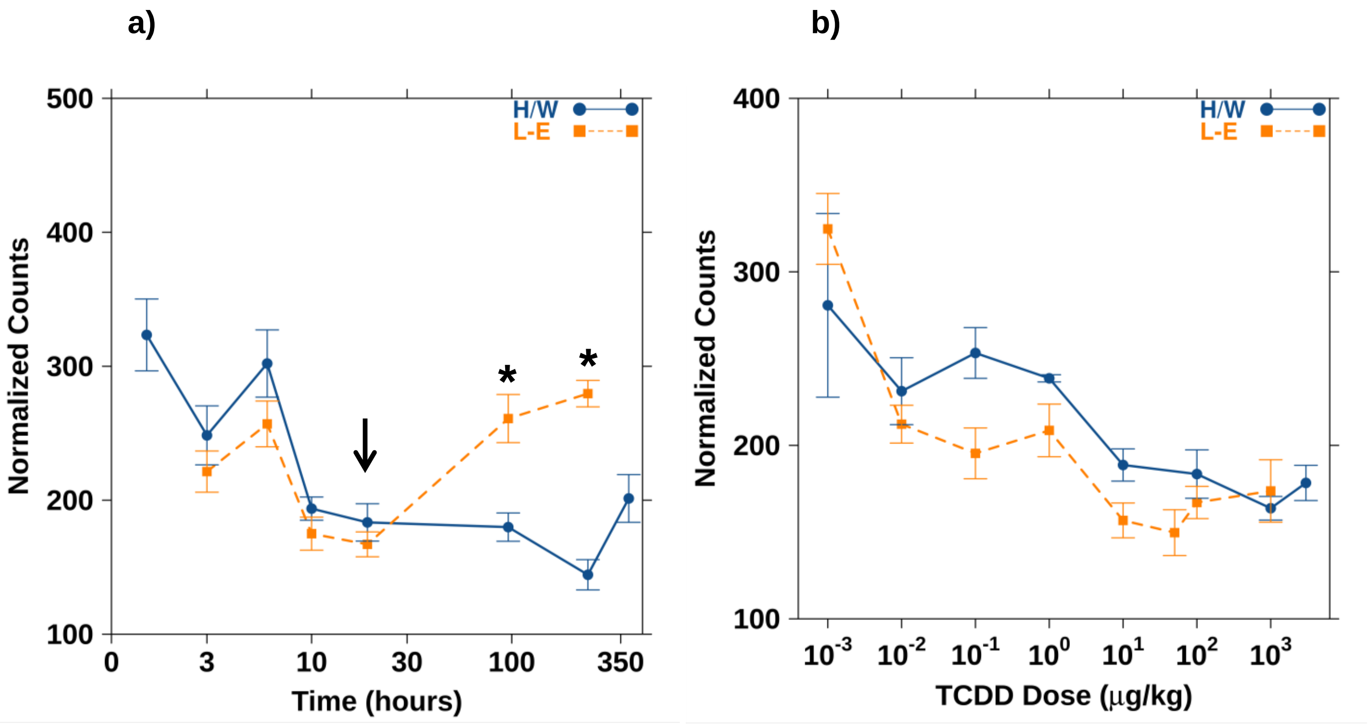

a)
b)
Normalized Counts
500
400
300
200
100
0
3
10
30
100
350
Time (hours)
H/W
L-E
*
*
Normalized Counts
400
300
200
100
10-3
10-2
10-1
100
101
102
103
TCDD Dose (µg/kg)
H/W
L-E

Fig. S14 *Exoc3*

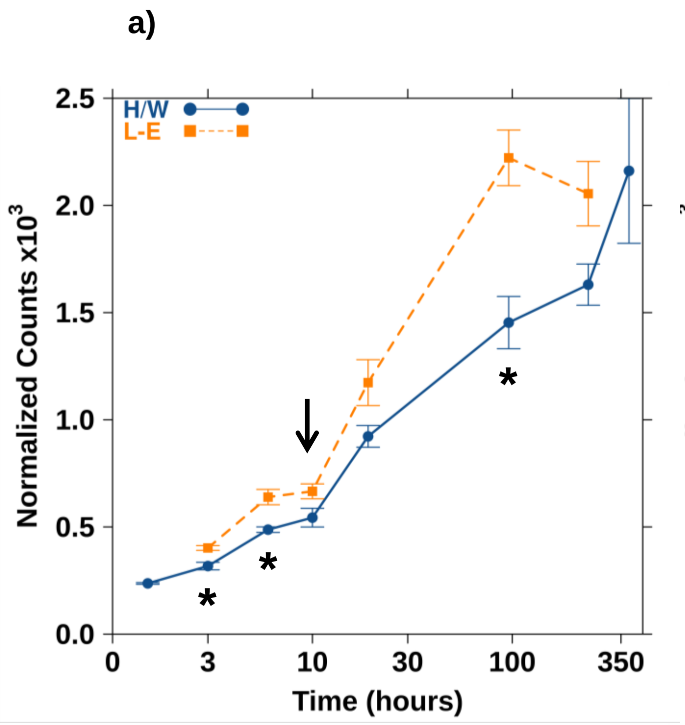

Fig. S15 *Ghr*

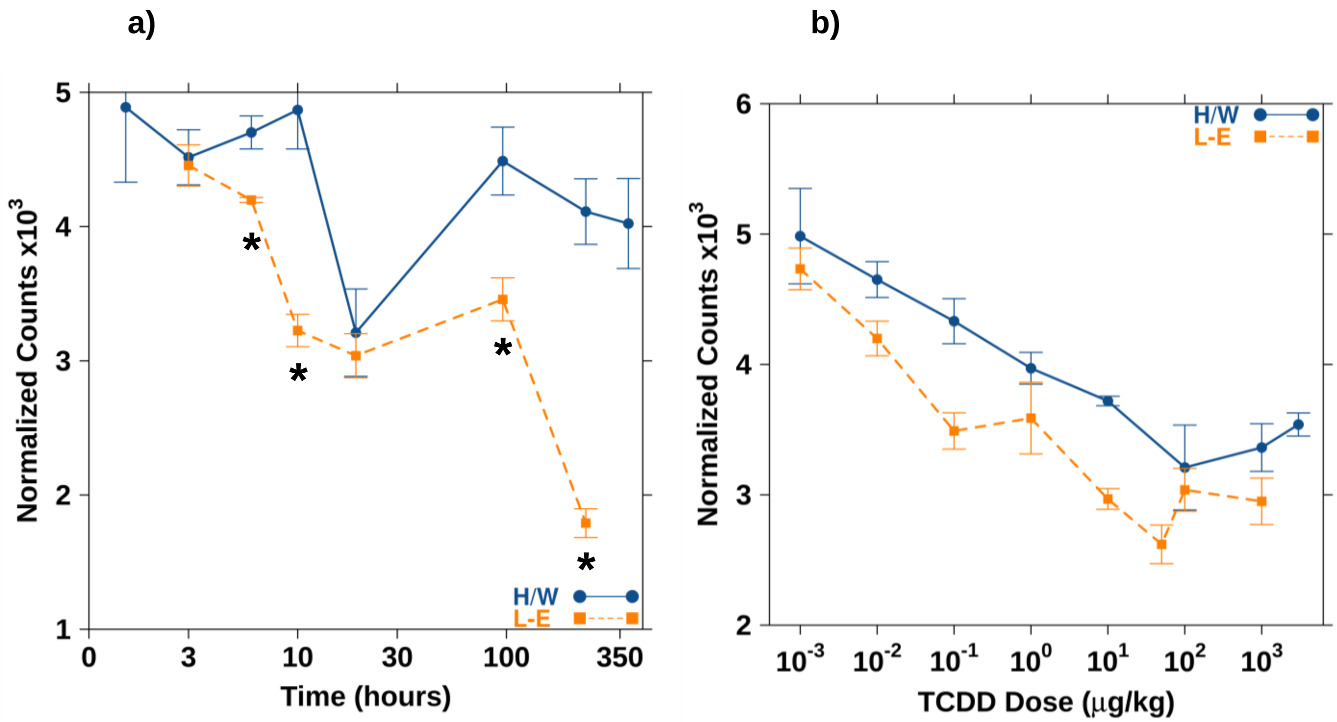
a)
b)
H/W
L-E
Normalized Counts x10^3
Time (hours)
0
3
10
30
100
350
1
2
3
4
5
*
H/W
L-E
Normalized Counts x10^3
TCDD Dose (μg/kg)
10^-3
10^-2
10^-1
10^0
10^1
10^2
10^3
2
3
4
5
6

Fig. S16 *Lasp1*

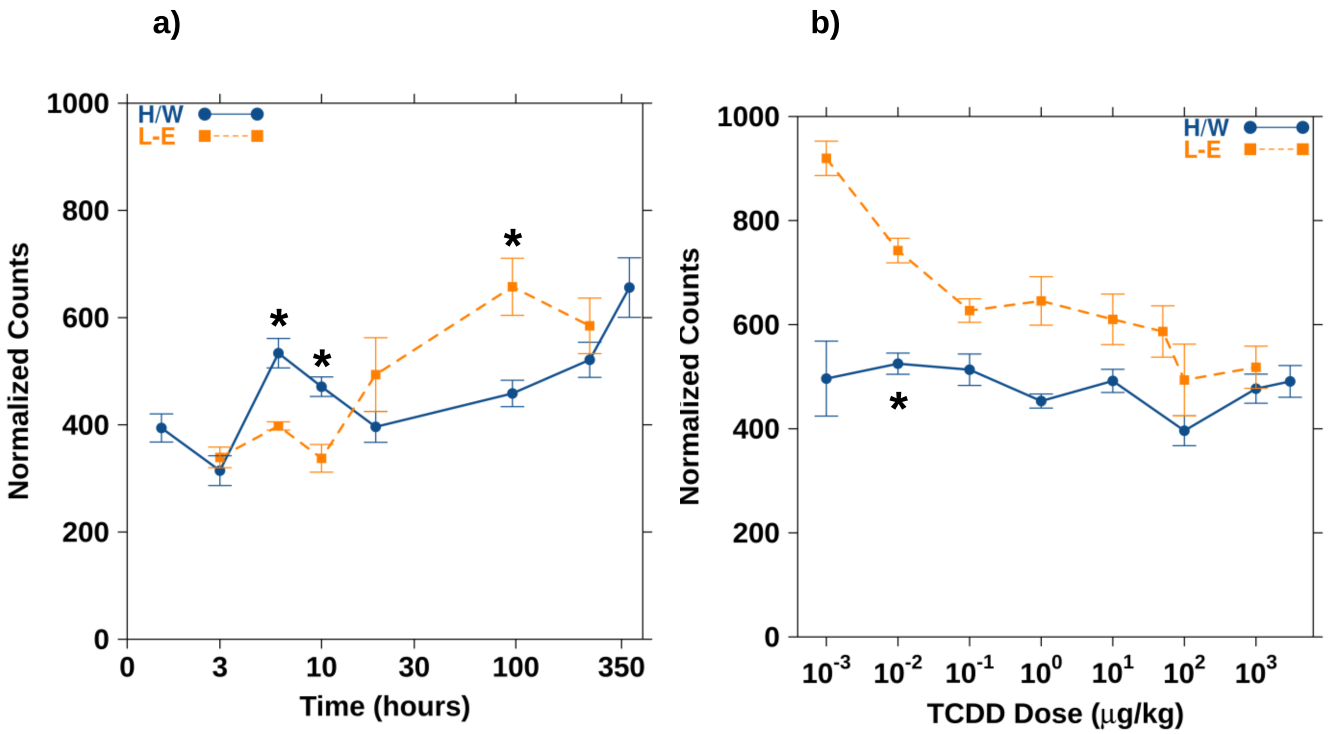

Fig. S17 *Neu1*

a)

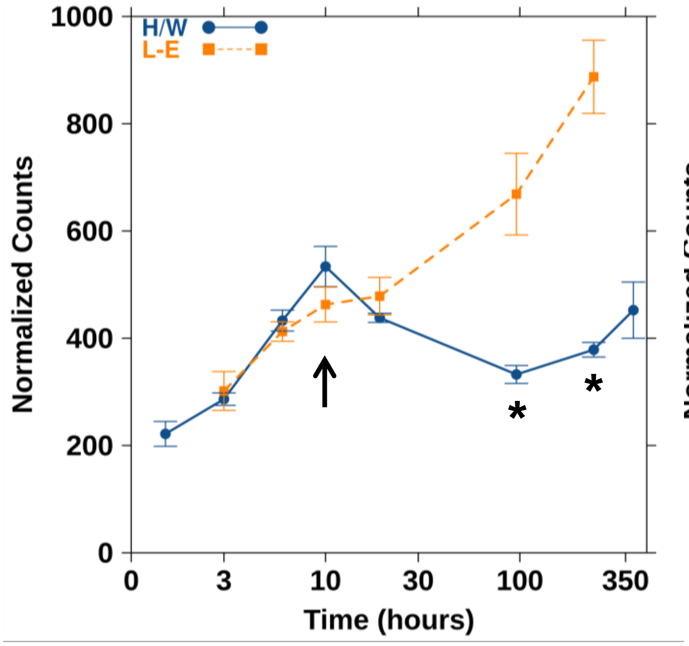

b)

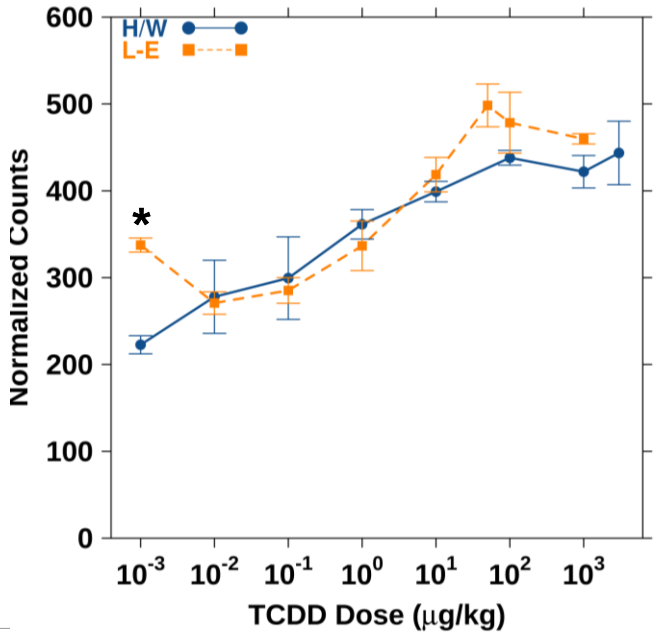

Fig. S18 *Perp*

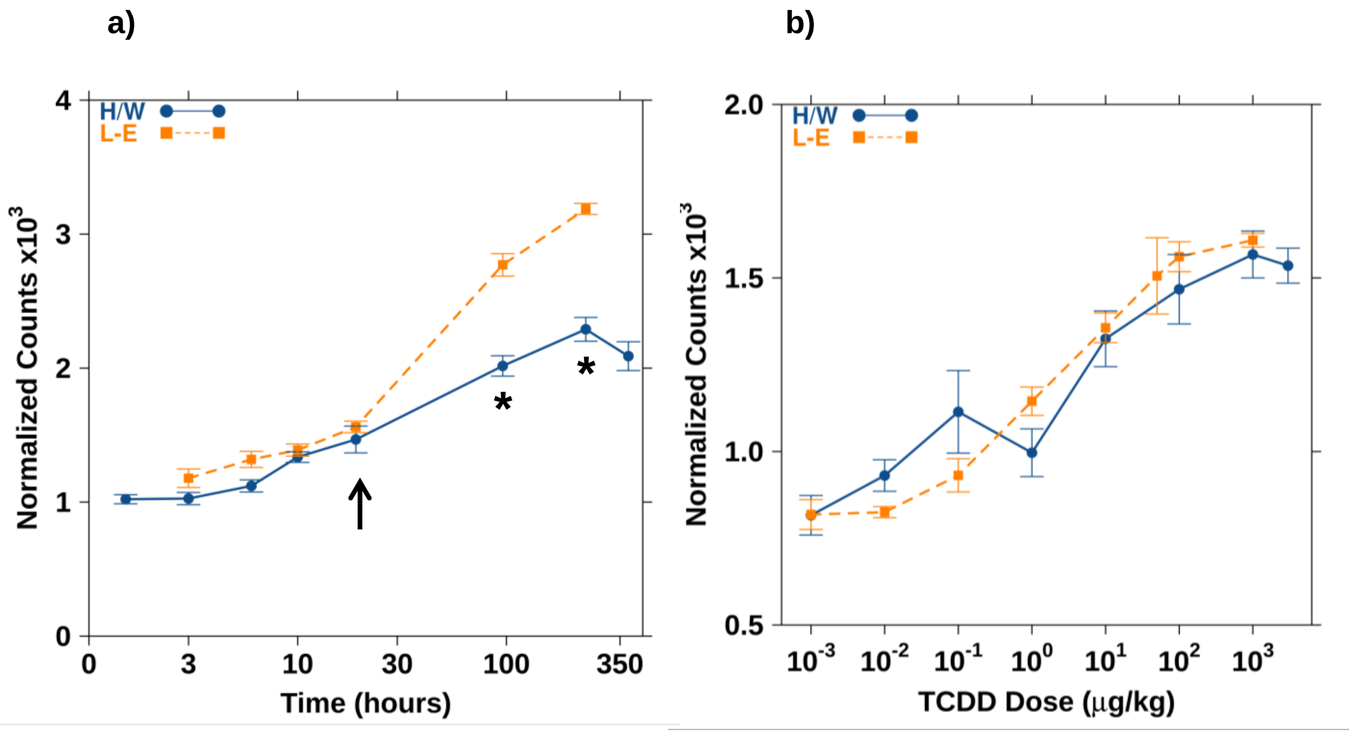

a)
H/W
L-E
Normalized Counts x10^3
0
1
2
3
4
0
3
10
30
100
350
Time (hours)
*
*
b)
H/W
L-E
Normalized Counts x10^3
0.5
1.0
1.5
2.0
10^-3
10^-2
10^-1
10^0
10^1
10^2
10^3
TCDD Dose (μg/kg)

Fig. S19 *Pmm1*

a)

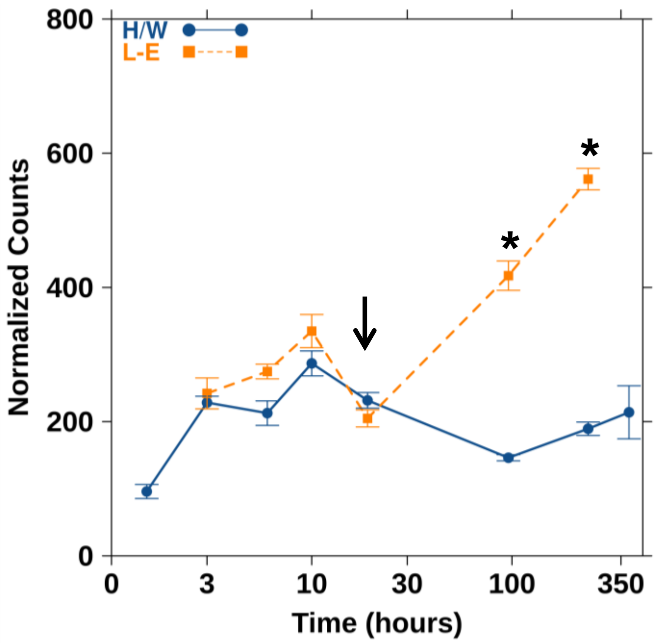

b)

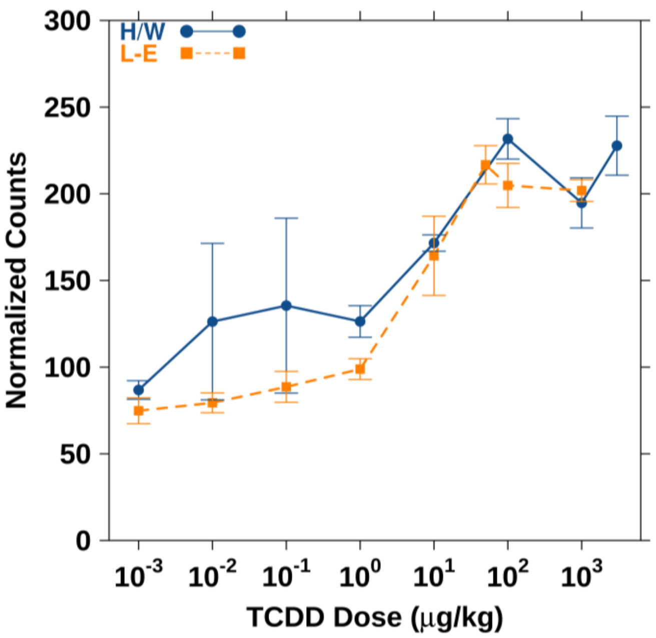

Fig. S20 *Psmb4*

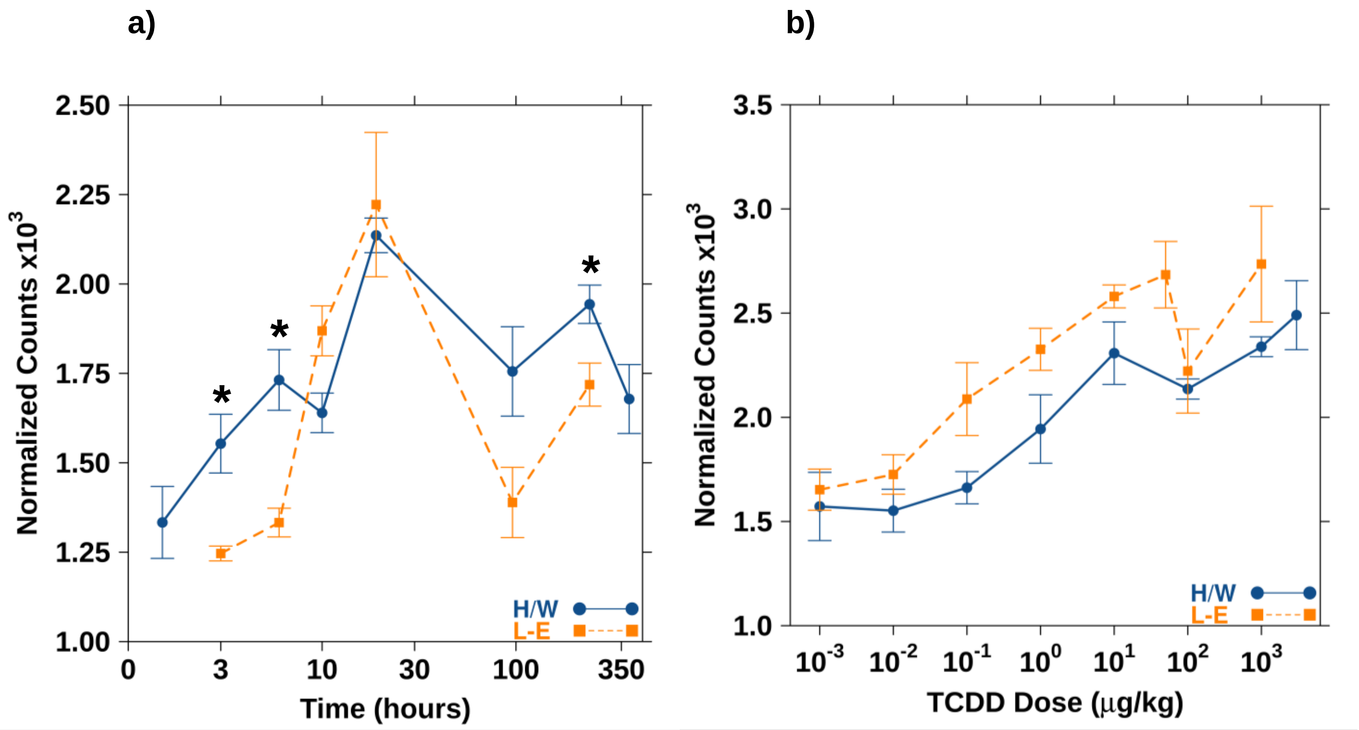

Fig. S21 *Sdc1*

a)

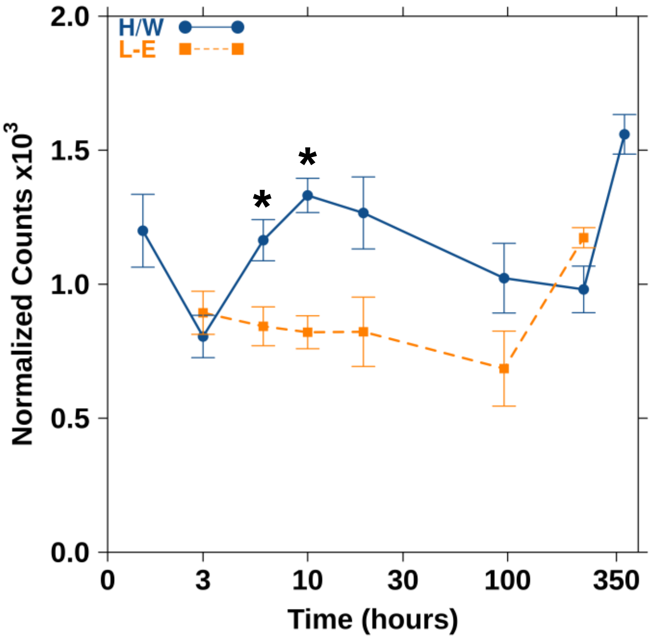

b)

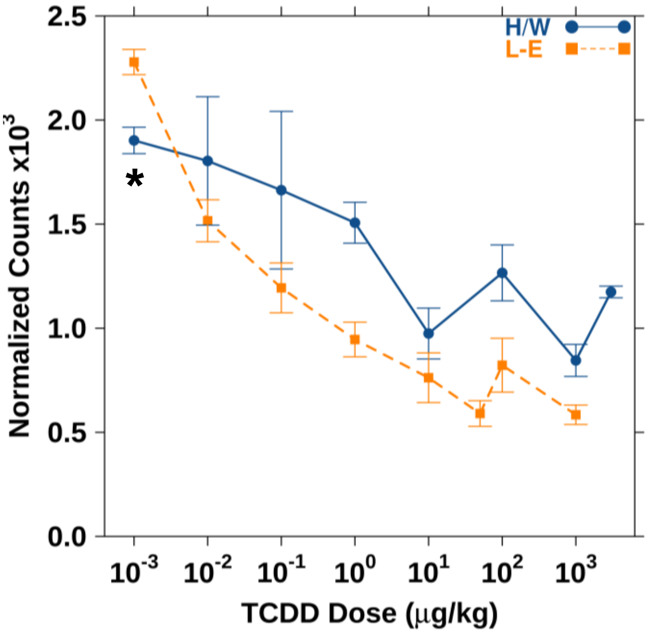

Fig. S22 *Srxn1*

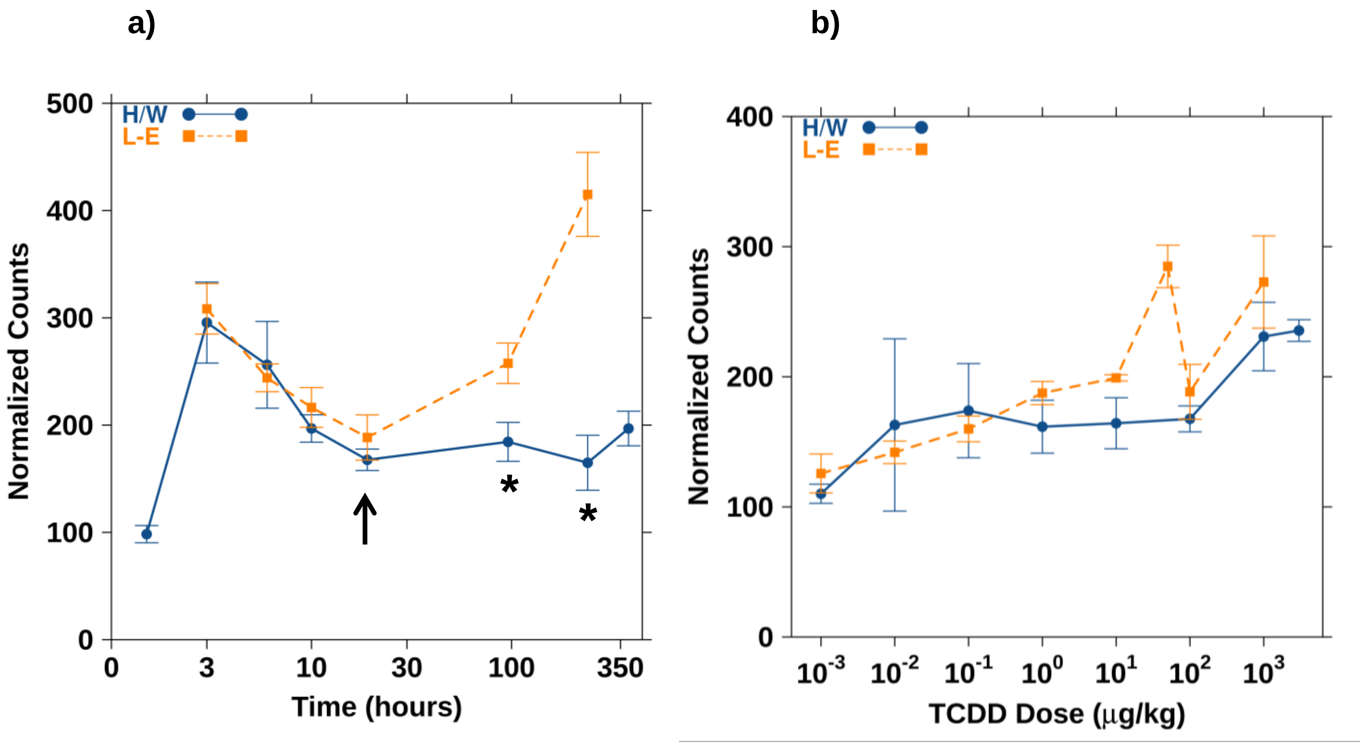

Fig. S23 Logistic curve fit for dose response data

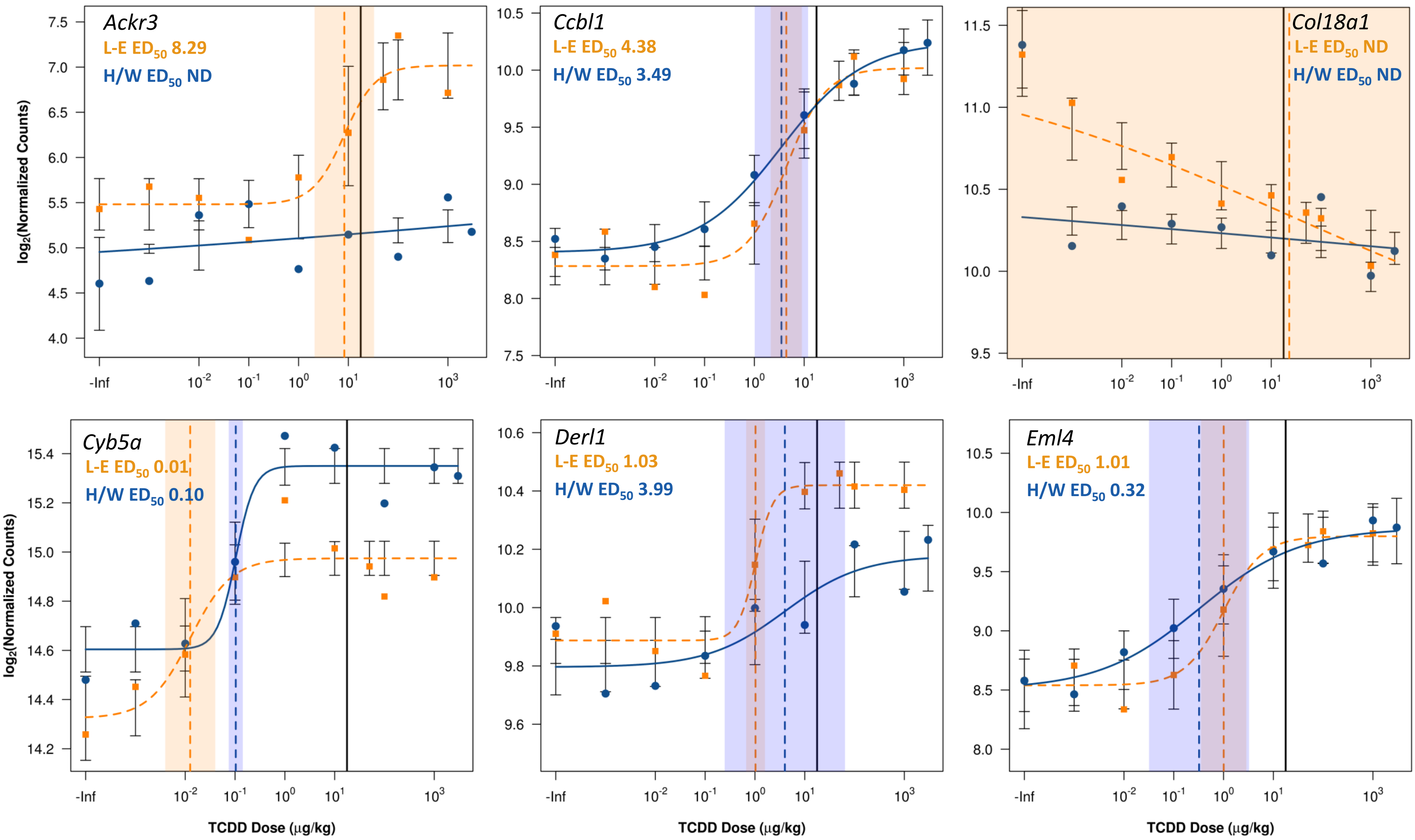

Fig. S24 Basal levels of expression for corn oil treated animals

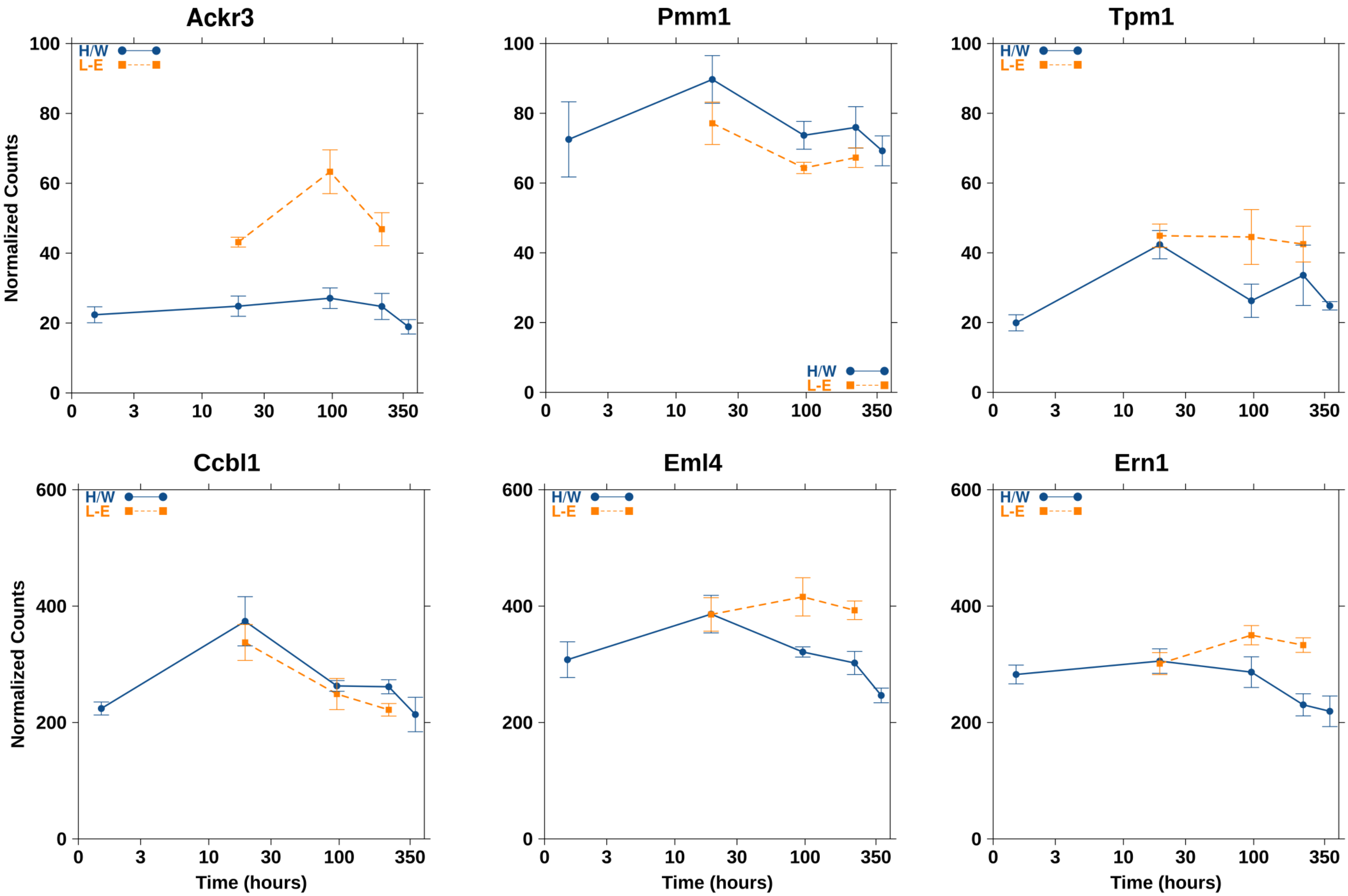

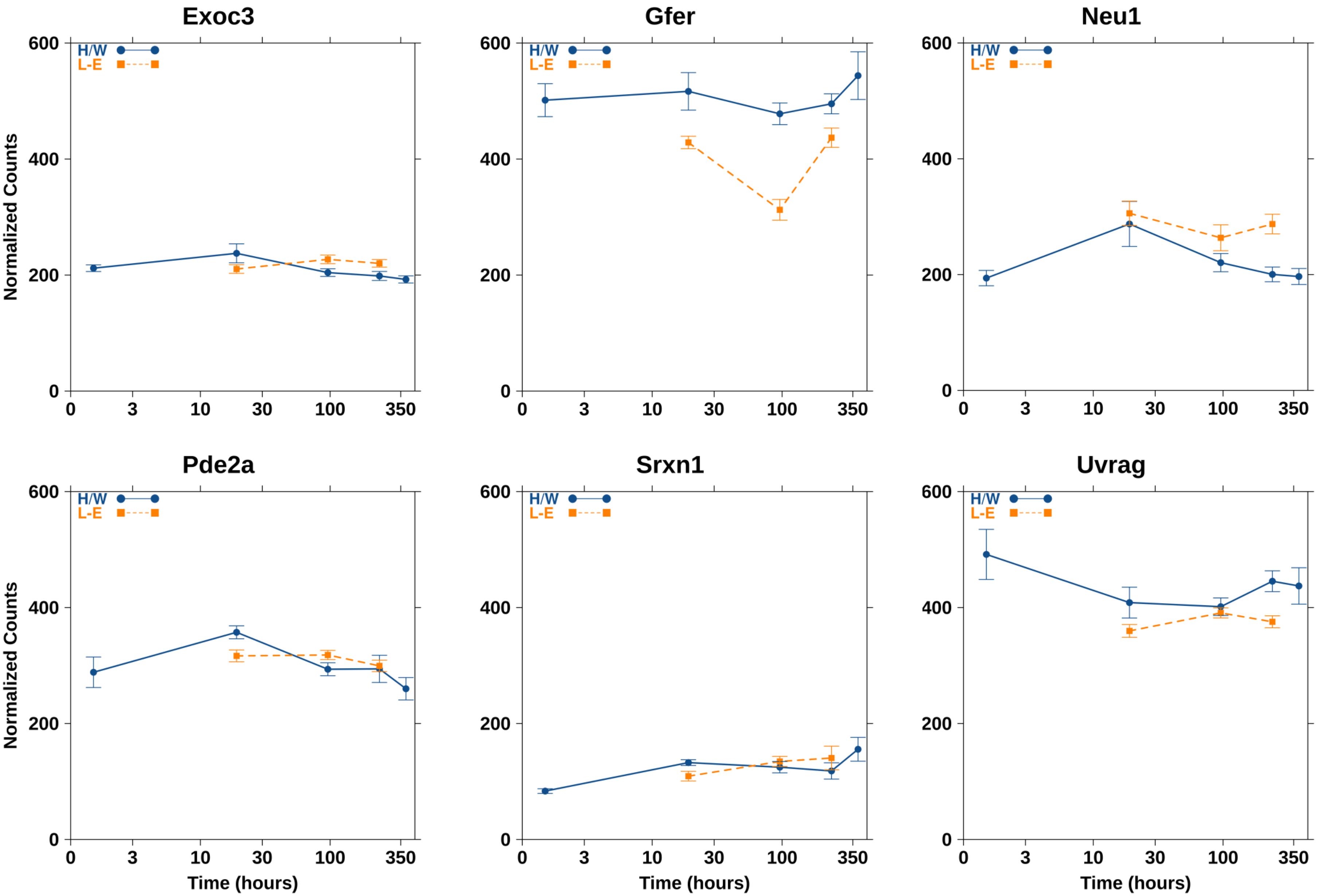

Exoc3
Gfer
Neu1
Pde2a
Srxn1
Uvrag
H/W
L-E
Normalized Counts
Time (hours)
0
200
400
600
0
3
10
30
100
350

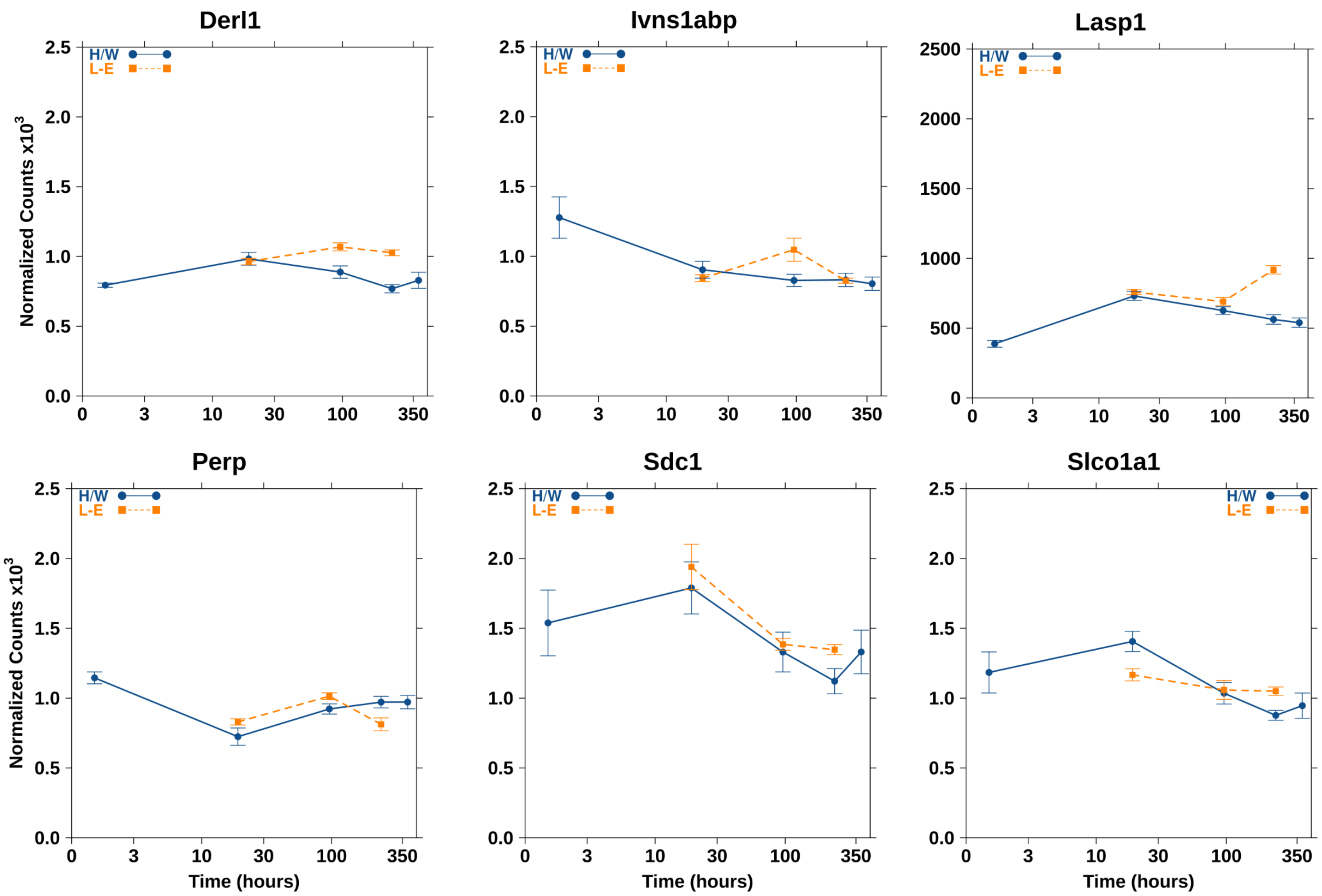

Derl1
Ivns1abp
Lasp1
Perp
Sdc1
Slco1a1
H/W
L-E
Normalized Counts x10^3
Time (hours)
0
3
10
30
100
350
0.0
0.5
1.0
1.5
2.0
2.5
500
1000
1500
2000
2500

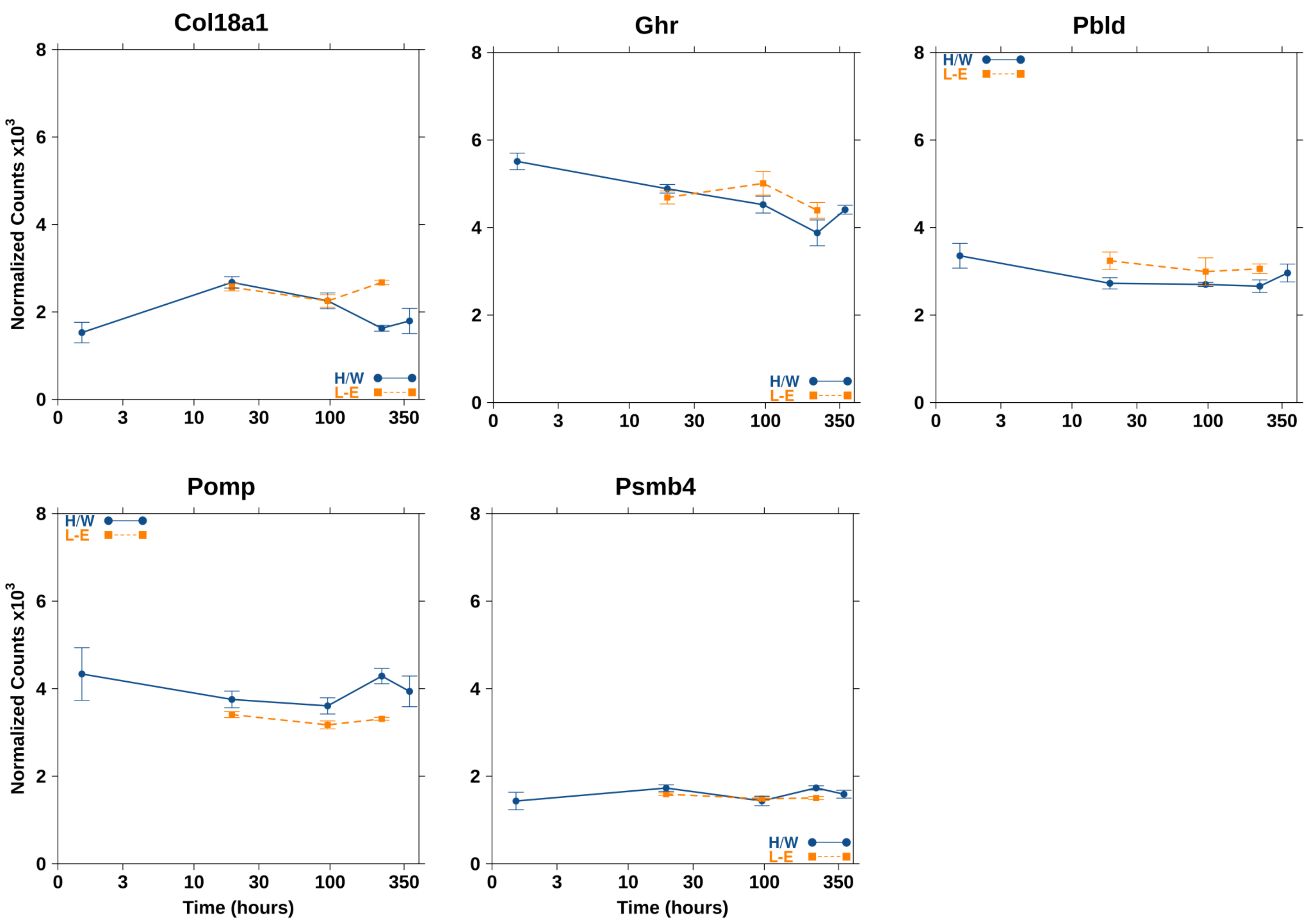

Col18a1
Ghr
Pbld
Pomp
Psmb4
Normalized Counts x10^3
Time (hours)
H/W
L-E
0
2
4
6
8
0
3
10
30
100
350

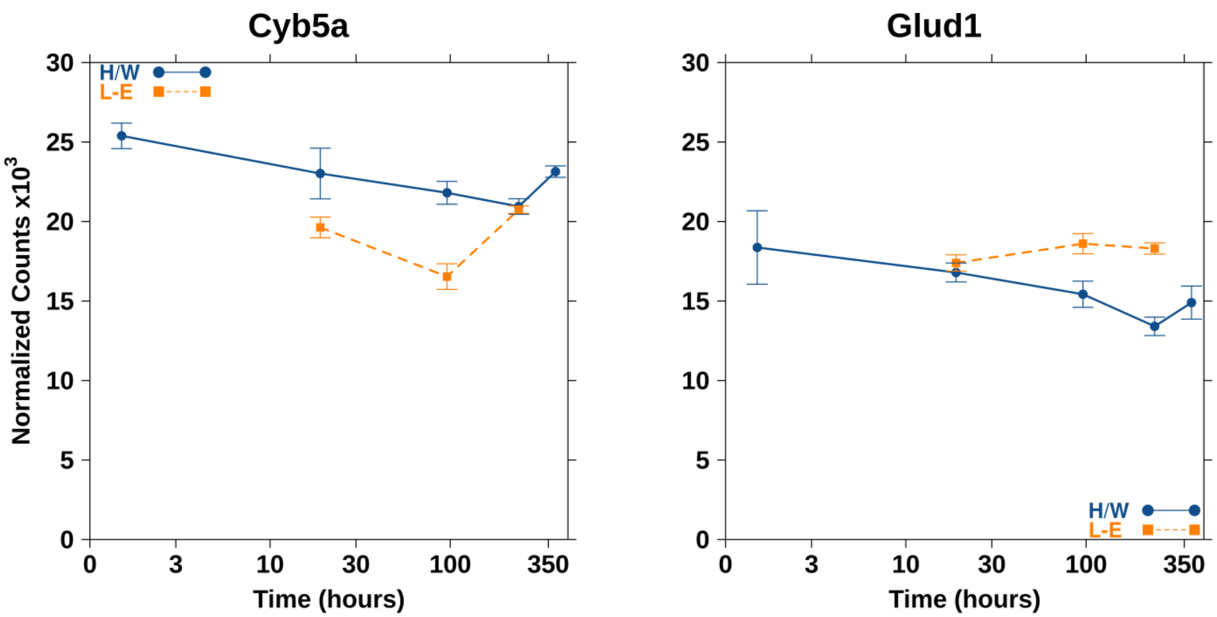
Cyb5a
Glud1
Normalized Counts x10^3
Time (hours)
H/W
L-E
0
3
10
30
100
350
0
5
10
15
20
25
30

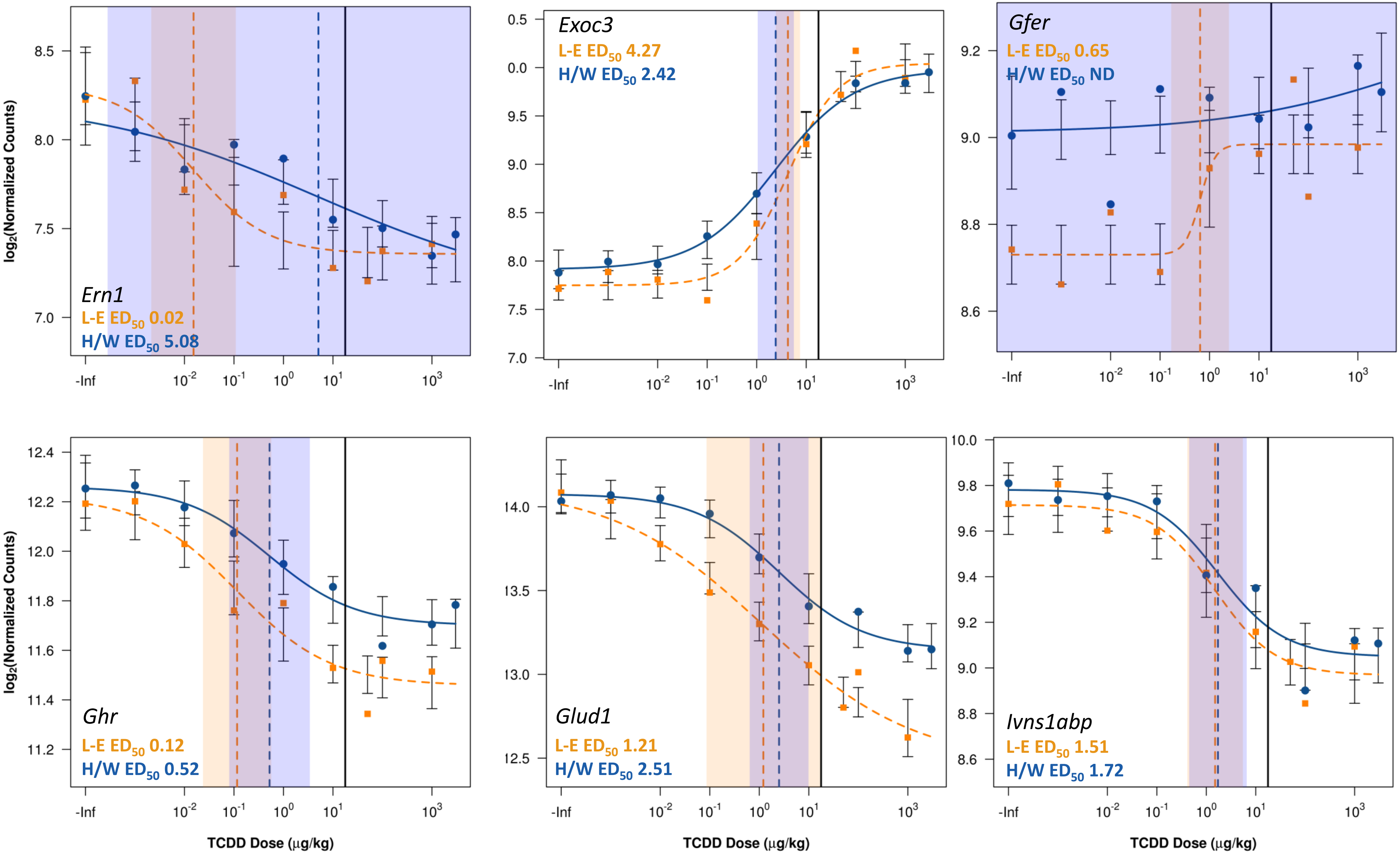
Ern1
L-E ED50 0.02
H/W ED50 5.08
Exoc3
L-E ED50 4.27
H/W ED50 2.42
Gfer
L-E ED50 0.65
H/W ED50 ND
Ghr
L-E ED50 0.12
H/W ED50 0.52
Glud1
L-E ED50 1.21
H/W ED50 2.51
Ivns1abp
L-E ED50 1.51
H/W ED50 1.72
log2(Normalized Counts)
TCDD Dose (µg/kg)
-Inf
10-2
10-1
100
101
103

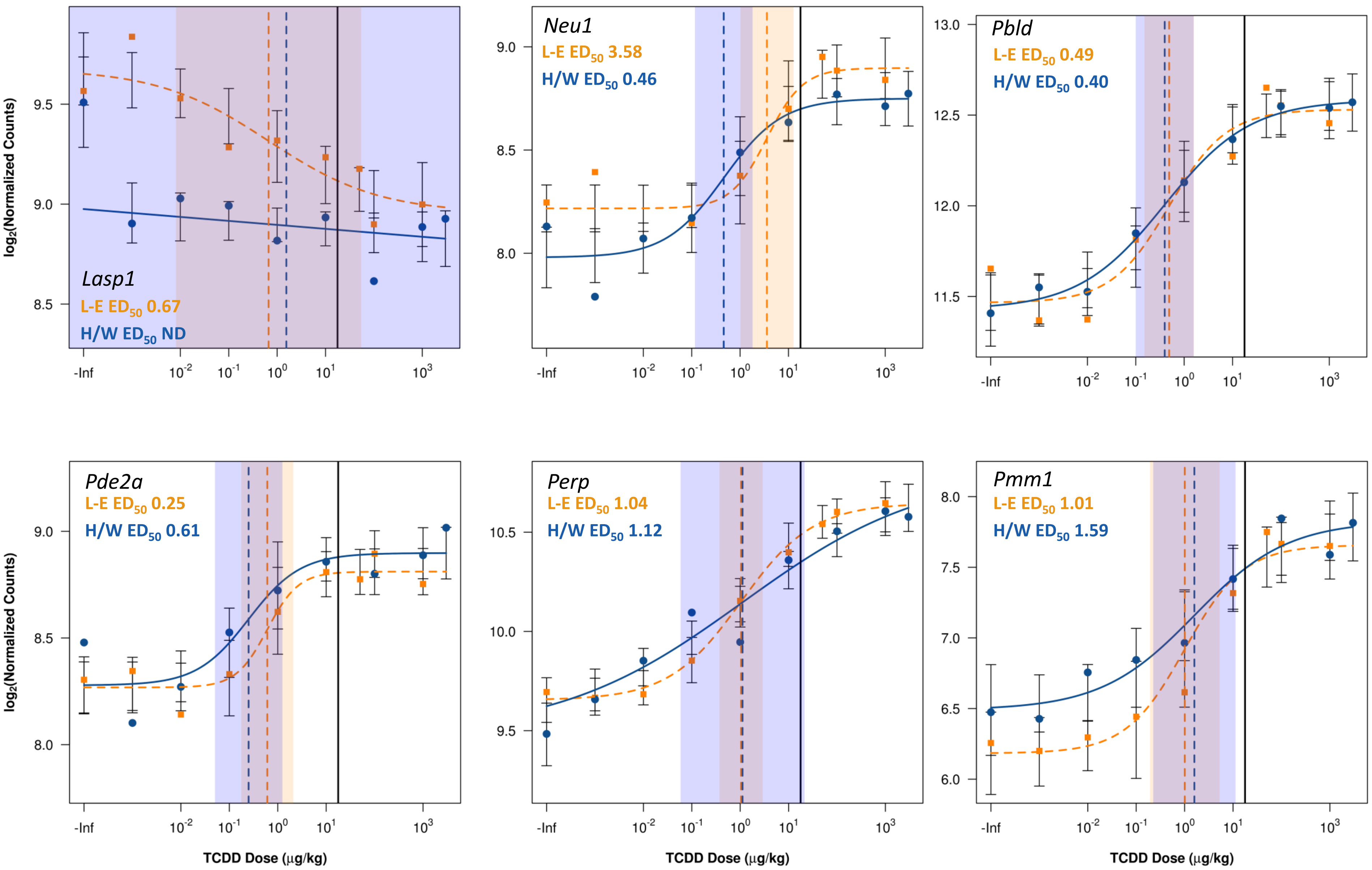

Lasp1
L-E ED_{50} 0.67
H/W ED_{50} ND
Neu1
L-E ED_{50} 3.58
H/W ED_{50} 0.46
Pbld
L-E ED_{50} 0.49
H/W ED_{50} 0.40
Pde2a
L-E ED_{50} 0.25
H/W ED_{50} 0.61
Perp
L-E ED_{50} 1.04
H/W ED_{50} 1.12
Pmm1
L-E ED_{50} 1.01
H/W ED_{50} 1.59
log_2(Normalized Counts)
TCDD Dose (μg/kg)
-Inf
10^{-2}
10^{-1}
10^{0}
10^{1}
10^{3}

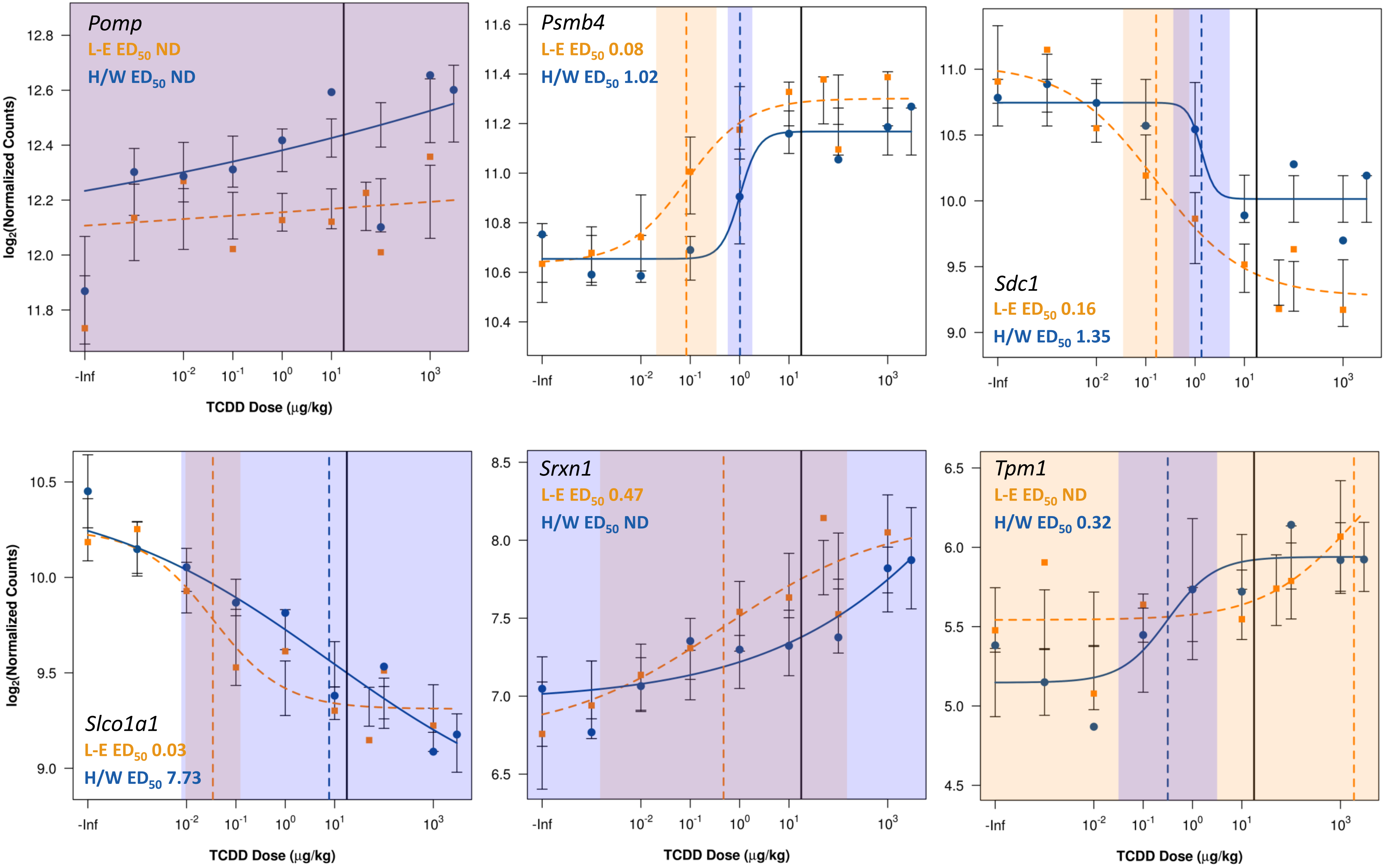

Pomp
L-E ED50 ND
H/W ED50 ND
Psmb4
L-E ED50 0.08
H/W ED50 1.02
Sdc1
L-E ED50 0.16
H/W ED50 1.35
Slco1a1
L-E ED50 0.03
H/W ED50 7.73
Srxn1
L-E ED50 0.47
H/W ED50 ND
Tpm1
L-E ED50 ND
H/W ED50 0.32
log2(Normalized Counts)
TCDD Dose (µg/kg)

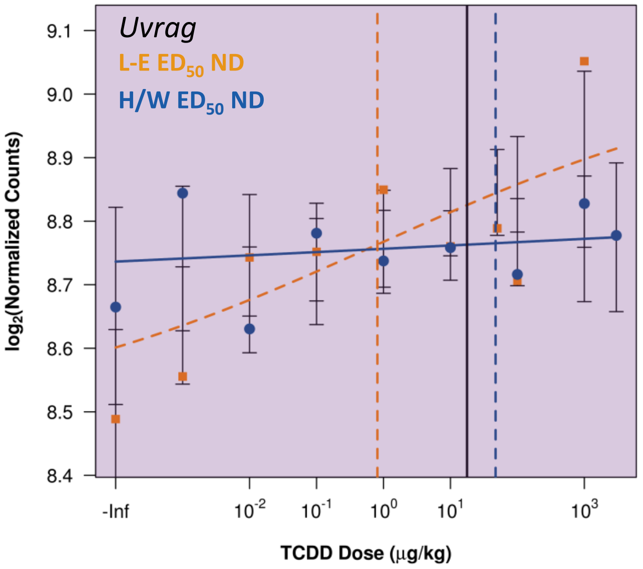

Uvrag
L-E ED50 ND
H/W ED50 ND
log2(Normalized Counts)
9.1
9.0
8.9
8.8
8.7
8.6
8.5
8.4
-Inf
10-2
10-1
100
101
103
TCDD Dose (μg/kg)
